# Review of Additively Manufactured Polymeric Metamaterials: Design, Fabrication, Testing and Modeling

**DOI:** 10.3390/polym15193858

**Published:** 2023-09-22

**Authors:** Abdulla Almesmari, Nareg Baghous, Chukwugozie J. Ejeh, Imad Barsoum, Rashid K. Abu Al-Rub

**Affiliations:** 1 Advanced Digital & Additive Manufacturing Center, Khalifa University of Science and Technology, Abu Dhabi P.O. Box 127788, United Arab Emirates; 2 Department of Mechanical Engineering, School of Engineering, Khalifa University of Science and Technology, Abu Dhabi P.O. Box 127788, United Arab Emirates; 3 Department of Engineering Mechanics, Royal Institute of Technology (KTH), Teknikringen 8, 100 44 Stockholm, Sweden

**Keywords:** architected materials, lattices, polymeric composites, additive manufacturing, mechanical characterization

## Abstract

Metamaterials are architected cellular materials, also known as lattice materials, that are inspired by nature or human engineering intuition, and provide multifunctional attributes that cannot be achieved by conventional polymeric materials and composites. There has been an increasing interest in the design, fabrication, and testing of polymeric metamaterials due to the recent advances in digital design methods, additive manufacturing techniques, and machine learning algorithms. To this end, the present review assembles a collection of recent research on the design, fabrication and testing of polymeric metamaterials, and it can act as a reference for future engineering applications as it categorizes the mechanical properties of existing polymeric metamaterials from literature. The research within this study reveals there is a need to develop more expedient and straightforward methods for designing metamaterials, similar to the implicitly created TPMS lattices. Additionally, more research on polymeric metamaterials under more complex loading scenarios is required to better understand their behavior. Using the right machine learning algorithms in the additive manufacturing process of metamaterials can alleviate many of the current difficulties, enabling more precise and effective production with product quality.

## 1. Introduction

The persistent quest of scientists for candidate materials and designs is attributed to the advent of cutting-edge technologies. With advances in material synthesis and additive manufacturing techniques, the fabrication of these materials and designs has become conceivable. In recent years, metamaterials emerged as one of the leading-edge technologies enabling the production of multi-functional structures on macro- and nano-scales. Generally, metamaterials are defined as artificially engineered materials mimicking nature-based architectures synthesizing extreme material properties that are rarely observed in bulk material form. Metamaterials, also known as lattice, architected or cellular materials/structures, are multi-functional materials executing numerous functions by virtue of tailored electromagnetic [1], optical [2], acoustic [3], thermal [4], and mechanical properties [5] for diversified purposes.

**Figure 1:**
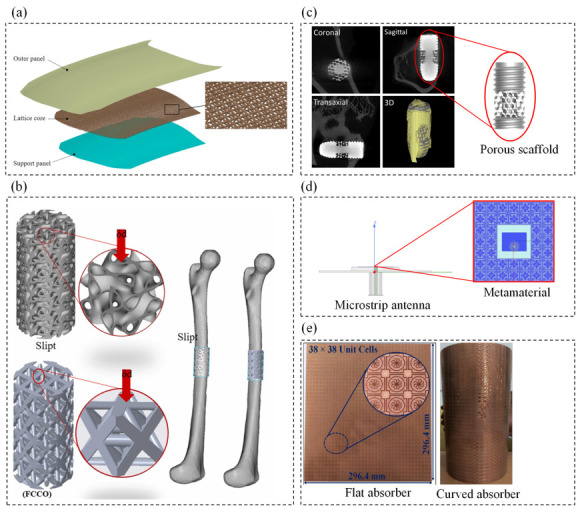
(**a**) Exploded view of lattice core sandwich hood (Reproduced with permission from [8]. Copyright 2018, Elsevier). (**b**) 3D models of the lattice design of slipt and face center cubic-octahedron (FCOO)/octet-truss on the femur bone (Reproduced with permission from [10]. Copyright 2022, Elsevier). (**c**) Micro-CT images of bone growth in the porous Ti6Al4V scaffold (Reproduced with permission from [12]. Copyright 2020, Elsevier). (**d**) 3D demonstration of microstrip antenna loaded with periodic metamaterials used for electromagnetic wave absorption (Reproduced with permission from [14]. Copyright 2021, IEEE). (**e**) Photographs of flat and curved metamaterial absorber sheets (Reproduced with permission from [15]. Copyright 2022, Wiley).

Lately, extensive investigations were conducted to explore the proficiencies of mechanical metamaterials in colonizing unexplored regions in the material space, such as ultrahigh strength-to-weight ratio, negative Poisson’s ratio, and extreme energy absorption capability [6]. Subsequently, different types of fields exploited the advantages of mechanical metamaterials. For example, in the automotive industry, Papetti et al. [7] utilized polyhedral structures made of Al_2_O_3_ ceramic as substrates for automotive catalysts. As depicted in Figure 1a, Yin et al. [8] proposed a double-curvature sandwich hood consisting of two fiber-reinforced composite panels and a pyramidal lattice core to improve the pedestrian protection performance of sandwich hoods. Furthermore, metamaterials in biomedical engineering are optimized in terms of mechanical, chemical, and biological properties as they interact with, and replace the function of certain host tissue [9]. For example, Oladapo et al. [10] introduced poly-ether-ether-ketone, and reduced graphene oxide (PEEK-rGO) scaffolds as potent bone implants for biomimetic heterogeneous bone repair. Figure 1b depicts the lattice design of slipt and face center cubic-octahedron (FCOO)/octet-truss on the femur bone. Additionally, Reyes et al. [11] demonstrated the capability of a polycaprolactone honeycomb structure scaffold in bone regeneration with suitable mechanical strength and stiffness. Along the same lines, osteointegration is the most detrimental process for a successful dental implant whereby jawbone cells grow over the implant to secure structural and functional connections [9]. In this regard, Xiong et al. [12] introduced a porous Ti_6_Al_4_V dental scaffold with high yield and fatigue strengths exhibiting favorable bone ingrowth and osteointegration. Figure 1c demonstrates micro-CT images of bone growth in the porous Ti_6_Al_4_V scaffold in coronal, sagittal, and trans-axial planes of the host bone. Due to their high strength-to-weight ratio and low coefficients of thermal expansion, polymeric composites appear to be promising for spacecraft skeletons. As a spacecraft experiences thermal cycling during its trajectory in an orbit, degradation of the composite mechanical performance would result in a catastrophic failure. Li et al. [13] studied the effect of vacuum thermal cycling on the out-of-plane compression and shear performance of polymeric composite sandwich panels containing pyramidal truss cores. Besides, many studies have shown the suitability of metamaterials for defense stealth technology as they can provide an excellent absorption of electromagnetic waves by configuring their size and topology. For example, Figure 1d illustrates a microstrip antenna loaded with a metamaterial absorber used for electromagnetic waves absorbance [14]. Other examples are depicted in Figure 1e which shows ultra-thin, conformal, triple band metamaterial absorbers [15]. Table 1 reports the application of different types of lattice materials involved in a variety of areas and highlights the investigated topology, base material, and physical property. As indicated, polymeric metamaterials have been recently involved in dentistry, bone implant, and spacecraft systems. The following sections will provide further details on the design/topology of the lattice materials considered.

**Figure 2:**
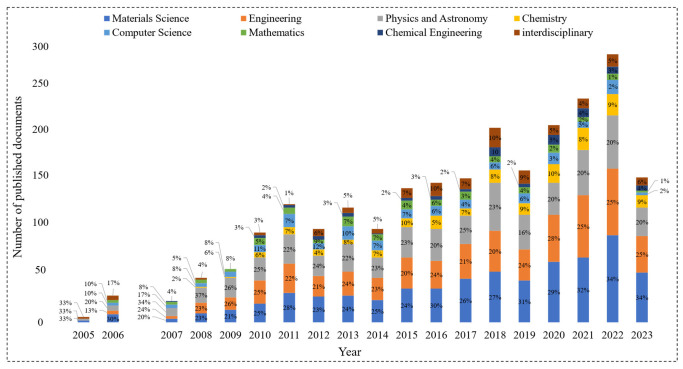
Database information obtained from Scopus using the keywords “metamaterials” and “polymers” (lastly updated on 12 July 2023).

According to the Scopus citation database, throughout the past two decades, a variety of subject areas exhibited an increasing interest in the fields of metamaterials and polymers as depicted in Figure 2 which describes the number of published documents (i.e., full research articles, reviews, conference papers, and book chapters) from 2005 up to date for different subject areas. Each bar represents the total number of published documents per year, where different colors indicate specific subject areas. The contribution to each subject area out of the total number of published documents is quantified as a percentage. It can be noticed that most of the published documents were related to material science, engineering, and physics subject areas. In fact, from 2009 onwards, these subject areas had contributed almost equally per year as they heavily interlaced in the fields of metamaterials and polymers. Note, that the interdisciplinary subject area consists of fields such as medicine, biology, pharmacology, and environmental sciences that displayed a fluctuating trend from 2010 and onwards. The citation database indicates a drastic increase in publications related to metamaterials from 2006 onwards. Interestingly, during the same period, the material science and physics communities were revolutionized by the works of Pendry et al. [18] and Leonhardt [19] that demonstrated the concept of cloaking which describes the process of shielding an object from view (i.e., invisible) using metamaterials by controlling the incident electromagnetic radiation. Subsequently, material scientists and engineers have been urging us to explore the physical, chemical, and mechanical properties of metamaterials.

The recent advancements in additive manufacturing and material synthesis allowed the integration of polymeric metamaterials in real-world applications. For example, Veerabagu et al. [20] reviewed the progress of polymeric auxetic metamaterials utilized in tissue engineering and medical devices, such as stents and sensors. Fan et al. [21] characterized different types of polymeric metamaterials according to their electromagnetic and acoustic features, for example, electromagnetic metamaterials made of photo-curable resin are functional in terms of electromagnetic cloaking, while acoustic and thermal metamaterial made of ABS and epoxy resin are functional in terms of acoustic absorption and ultralow thermal conductivity, respectively. Furthermore, Al Mesmari et al. [22] characterized different types of mechanical metamaterials involved in impact absorption and load-bearing applications. However, there is still an apparent gap in categorizing the current polymeric metamaterials in terms of their mechanical properties, whether it is due to the various topologies, printing techniques used to manufacture the parts, the polymeric materials used, etc. Thus, the main aim of this review is to categorize the mechanical properties of existing polymeric metamaterials from the literature, to act as a reference for future engineering applications. In order to build on that, the present review includes a detailed collection of recent research on the design (Section 2), fabrication (Section 3) and testing of polymeric metamaterials (Section 4).

## 2. Design of Polymeric Metamaterials

**Figure 3:**
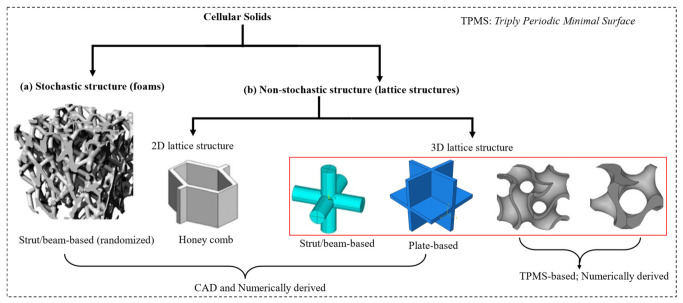
Classification of various types of lattice structures or cellular solids. (**a**) Stochastic lattices and (**b**) periodic lattices.

Metamaterials are usually arranged in a periodic network of structural elements or repeating patterns. This network of lattices exists on a wide range of scales, from the nanoscale to the macroscale, and is now a candidates for design in additive manufacturing. Importantly, the metamaterials’ effective working properties, such as their mechanical capabilities, can be modified by engineering the macro units to form specific microstructures with desired functional responses. Figure 3 illustrates examples for each major and subclass of lattice materials whose architectural multifunctional properties have been extensively explored in the literature [23,24,25,26]. It is worth noting that some lattices use the same recurring unit cell through the entire latticing bounds, and are referred to as periodic lattices (Figure 3b). The alternative form is for the cells to be randomly connected across the domain and are referred to as stochastic lattices (Figure 3a). Hence, this section reviews various categories and subclasses of popular lattice structural designs used in the fabrication of multifunctional metamaterials, and their design strategies. It also provides mathematical relations that can be used to predict the lattices’ topological properties such as relative density, surface area density (or surface area-to-volume ratio) and cell size using the design parameters.

### 2.1. Comb-Based Metamaterials

**Figure 4:**
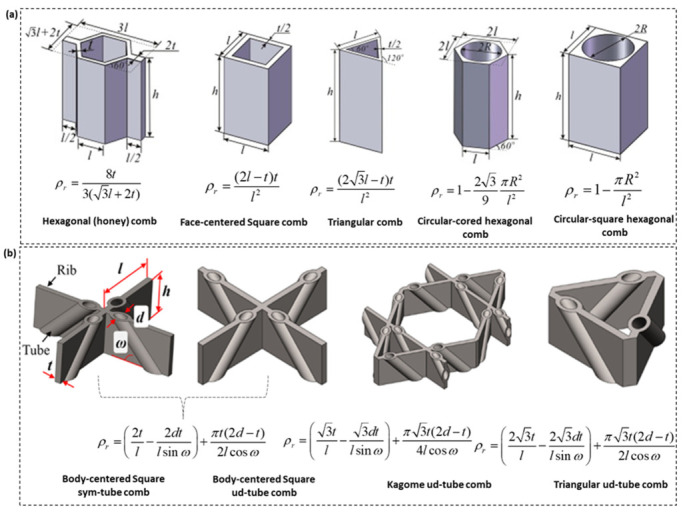
Comb lattice architectural designs. (**a**) Mono-topology unit cell shapes (Reproduced with permission from [29]. Copyright 2015, Elsevier) (**b**) Hybrid unit cell shapes with tubular structures for mitigating shear buckling effect in comb lattice structures (Reproduced with permission from [28] Copyright 2018, Elsevier). The figure also shows mathematical relations used to predict the relative density of the comb lattice structures as a function of extrusion length and plate thickness. The terms *t*, *h*, *l* and ω in the images mean thickness, depth, span length of the comb lattice, and rotation of tubular reinforcement, respectively.

A comb, extrusion, or 2D lattice structure is a collection of hollow unit cells made of thin vertical walls (Figure 4). The thin walls can be arranged differently to form specific 2D shapes (or topologies) which can substantially influence the mechanical performance of the metamaterial. Several comb lattice structural designs have been proposed by researchers, many of which are inspired by natural and biological features. Figure 4a shows basic comb lattice designs developed from arranging plates or sheets following a particular shape, for example, hexagonal, square, triangular, and combinations of these to form unit cells which can be repeated in two dimensions. Comb lattices are mostly closed cells and their sizes range from tens of micrometers to millimeters depending on the application. A major factor that has limited the mechanical performance of comb lattices, such as those presented in Figure 4, is out-of-plane bending deformations due to the anisotropic behavior of comb lattices [27]. Due to the obvious elastic nature of polymeric and bio-honeycomb structures, their mechanical behaviors differ dramatically from those of conventional honeycomb solids, such as the entirely reversible transition in buckling instability and the peculiar negative Poisson’s ratio [27]. Xu et al. [28] designed reinforced comb lattices with hollow tubes having different inclinations and rotations along shear planes in crystal structures, as shown in Figure 4b. Their designs showed to effectively mitigate issues with the buckling behavior of comb lattices in large deformation compression loading, which becomes significant as the comb lattice’s relative density increases. Herein, the relative density of lattices $\rho_{r}$ is calculated from dividing the lattice’s density $\rho_{l}$ to that of the solid $\rho_{s}$. Figure 4a,b show the mathematical relationship that can be used to predict the relative density of popular mono-topology and hybrid comb lattice structures [28,29]. Except for the hexagonal comb (or honeycomb) lattice structure, the relative density of the other comb lattices shows a direct quadratic relationship with sheet or plate thickness *t* and an inverse relationship with the span length *l* as presented in Figure 4a. Lattice truss structures with open-cell honeycomb topologies, such as those presented in Figure 4a, have emerged due to topology design and fabrication improvements. 

### 2.2. Strut-Based Metamaterials

Strut-based (or truss) lattices are a class of three-dimensional lattice structures. These structures, unlike comb lattice structures, are made of several rod-like formations that are joined in various orientations to produce the various unit cells of the lattice [30]. If the number and orientation of struts, and the number of nodes connecting the struts are not constrained, various strut-based lattice topologies can be developed and constructed within a given volume, allowing for the design of strut-based metamaterials with tunable physical and mechanical properties for engineering applications [31,32].

**Figure 5:**
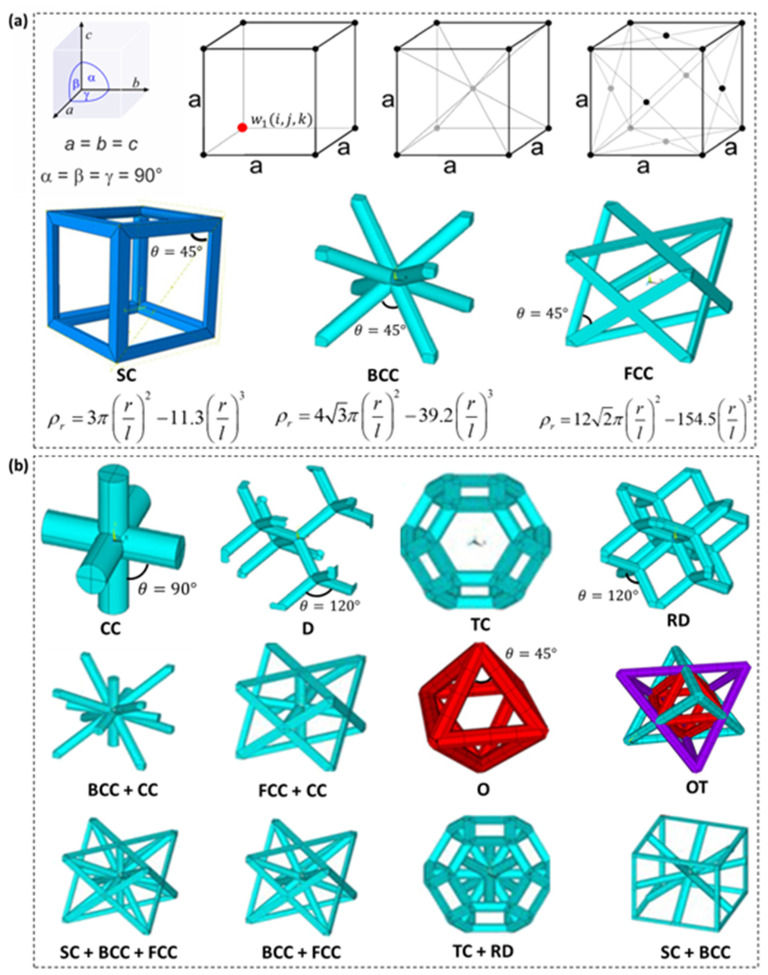
Illustration of various strut-based lattices. (**a**) Mono-topology strut-based architectures inspired by the Bravais lattice [35,37] (**b**) Other complex strut-based lattice forms [34,37]. The figure also shows mathematical relations used to predict the relative density of the comb lattice structures as a function of extrusion length and plate thickness. The terms *t*, *h*, *l* and ω in the images mean thickness, depth, span length of the comb lattice, and rotation of tubular reinforcement, respectively.

Several methods for designing strut-based lattice structures within an enclosure have been proposed by researchers, the most common being a cubic circumvallation. The most convenient method, however, is to create the struts around an axis connecting two nodes. A strut connects the nodes at strategic points in space. Inherently symmetric crystals can take on a variety of shapes. By rotating the crystal angle around a defined axis, an atomic arrangement identical to the native configuration ‘Bravais lattice’ could be obtained. Depending on the application, two or more of these atomic networks may be combined to improve the overall mechanical properties of the cellular material, which is a common practice in designing cubic lattices [31,33,34,35,36,37]. To fundamental strut-based lattices such as Simple Cubic (SC), Body-Centred Cubic (BCC) and Face-Centred Cubic (FCC) shown in Figure 5a (inspired by the Bravais lattice), the following is required; first, the spatial points, $w_{n}(i_{n},j_{n},k_{n})$ where *n* is an integer, to create nodes following crystal systems is required such that the lengths *a, b* and *c* are equal and have orientations of α, β, γ=90°. Then, struts with a specific radius *r* are constructed with terminals connecting at least two nodes in the Bravais lattice. Figure 5a shows some examples of lattice structures created through this simple design procedure, and much more can be designed by increasing the number of nodal points, number of struts and strut-orientations within a confined volume. Typical examples are the Truncated Cuboctahedron (TD), Diamond (D) and Rhombic Dodecahedron (RD) presented in Figure 5b. Moreover, other strut-based lattices are formed by hybridizing two mono-topologies; for example, the Octet-Truss (OT) lattice is formed by combining the Octet (O) topology and trusses, while the FCC+SC lattice is constructed by combination the FCC topology and SC topology. Similarly, more examples can be seen in Figure 5b. In addition, Figure 5a provides mathematical relations that can be used to predict the relative density of fundamental strut-based lattices (e.g., SC, FCC and BCC). These equations can serve as a basis for determining the relative density of hybrid strut-based lattices such as SC+FCC, SC+BCC, FCC+BCC and SC+BCC+FCC. Typically, the mathematical relations are determined by varying the strut radius *r* and length *l* and regressing the curve that relates relative density to *r*/*l* ratio. 

### 2.3. Plate-Based Metamaterials 

**Figure 6:**
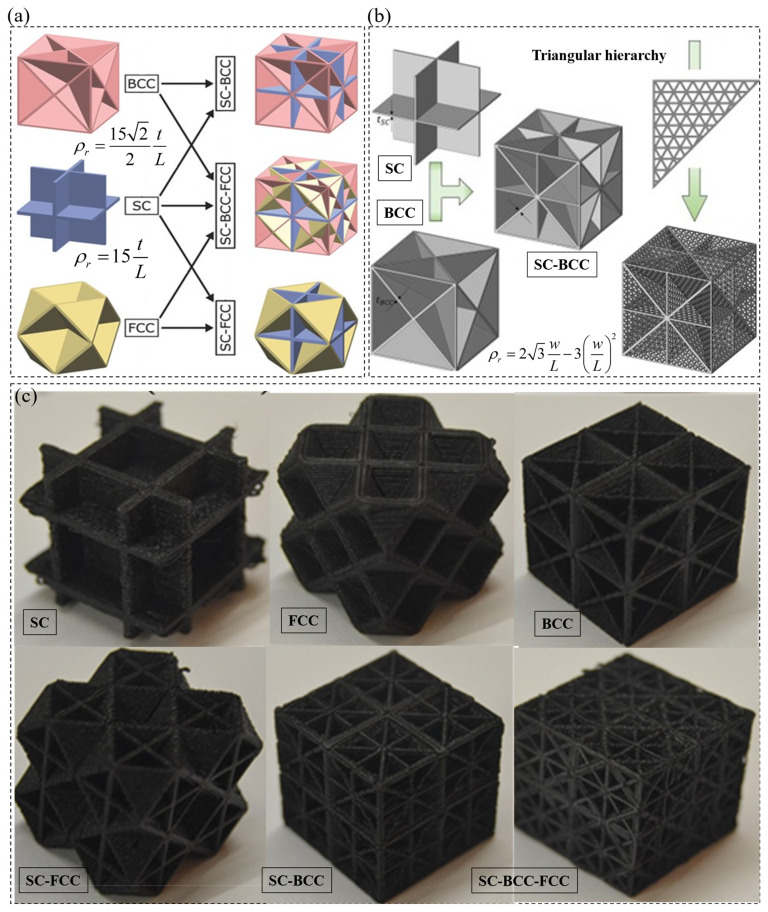
(**a**) 3D CAD designs of elementary and hybrid plate lattices (Reproduced with permission from [38]. Copyright 2018, Wiley), (**b**) 3D CAD designs of hierarchical plate lattice [41] and (**c**) 3D printed polymer-based plate lattices [40].

Plate lattices (Figure 6c) are a novel emergent class of cellular solids that approach the theoretical limitations of the stiffness of porous materials [38,39]. They have a significantly higher shear modulus compared to comb and truss lattice structures at the same relative density [38,39,40]. Plate lattices are constructed by placing plates along the various closest-packed crystal planes with normal vectors rotated at 45° and/or 90°, similar to truss lattice structures. Consequently, several plate lattice topologies with isotropic properties can be realized for better mechanical performance. Figure 6a shows most investigated mono-topology plate lattice architectures such as SC, BCC and FCC, which are elementary but non-isotropic plate architectures that can be combined to form isotropic plate lattice architectures such as SC-BCC, SC-FCC and SC-BCC-FCC. In order to obtain elastic isotropy, the mixing ratio of the SC and BCC/FCC elementary topologies in terms of solid volume must be equal to 1:4 to form SC-BCC or SC-FCC plate lattice structures, respectively. This can be achieved by fixing $\frac{t_{BCC}}{t_{SC}}=\sqrt{2}$, where t is the plate thickness [38,41]. The mathematical expressions relating the relative density of SC, BCC and FCC plate lattices as a function of plate thickness are provided in Figure 6a. The plate lattices shown in Figure 6a are closed-cell structures, meaning the cells are entirely enclosed by their walls. Figure 6b illustrates the process for creating hierarchical isotropic plate lattice (e.g., SC-BCC) using micro-triangular structures to create the hierarchy, along with a mathematical relation to predict its relative density in terms of the width of the triangulated truss w and unit cell size L, respectively [41]. 

### 2.4. TPMS Metamaterials 

**Figure 7:**
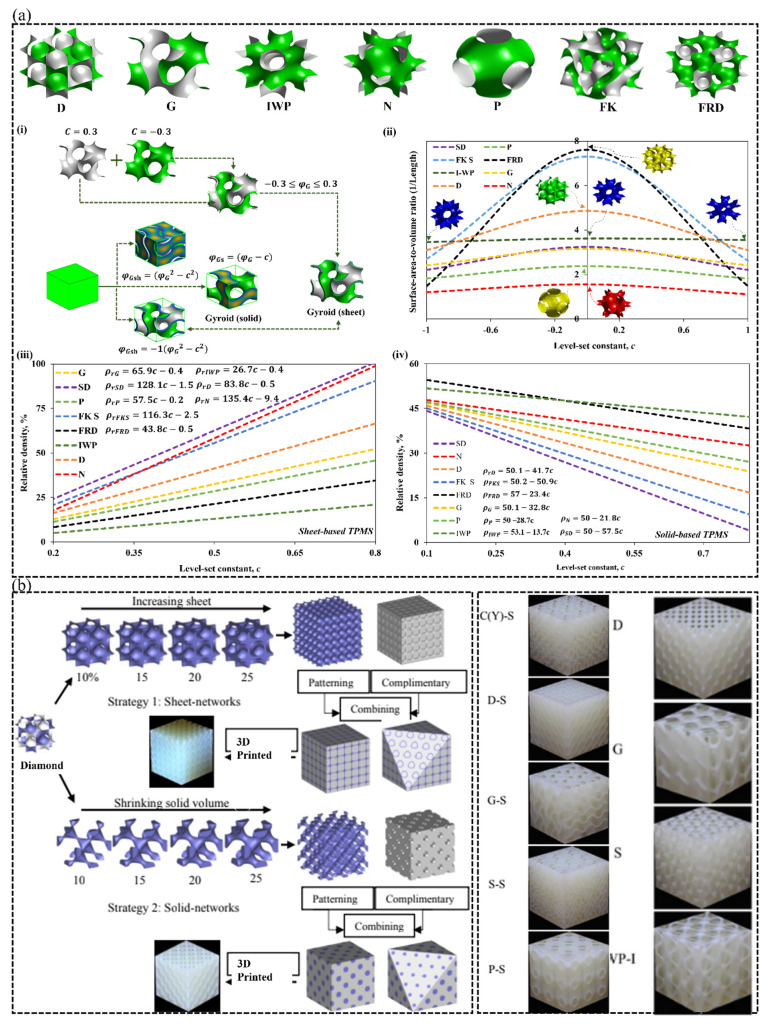
(**a**) Illustration of how sheet-based and ligament-based TPMS lattices can be designed, implicitly, along with plots that show the relationship between the parameters t and c, and relative density and surface area-to-volume ratio for selected TPMS lattice types, respectively. (**b**) Demonstration of the printability of these intricate structures using polymer-based additive manufacturing process and base material. Illustration of; (i) how sheet-based and ligament/solid-based TPMS architectures are constructred through implicity strategies, (ii) relationship between surface area-to-volume ratio of unit-cell sheet-based TPMS with variation in level-set parameter, (iii,iv) relationship between relative density and arbitrary parameter for sheet-based and ligament/solid-based TPMS architectures, respectively.

Surface-based porous structures are commonly used to describe lattices generated by trigonometric equations. The shape, size, and density of the 3D structure are controlled by their respective level-set equations governing their topology. Following this approach, researchers and mathematicians have developed several surface-based lattice structures with distinct and multifunctional properties over the years [23]. Triply periodic minimal surfaces (TPMS) are a subset of self-supporting surface-based lattices and are defined by mathematical equations, the most common of which is the level-set approximation equation. The level-set approximation equation φ*,* as previously stated, is frequently used to generate TPMS-based lattices. φ is based on the following Fourier series expression, ψ(r):

(1) $$\psi (r)\approx \sum_{h}F(h)\cos\left[2\pi h.r-\vartheta (h)\right]=0$$

where r is the normal vector to the surface, h is the reciprocal vector, ϑ(h) is the phase shift between two nodal points, and F(h) describes the wave amplitude for the vector k. Schwartz Primitive (P), Fischer Koch S (FK), Schoen F-Rhombic Dodecahedral (FRD), Schwartz Diamond D, Schoen Gyroid, Schoen I-Wrapped Package (IWP), Neovius (N), and Standard Diamond (SD; obtained by rotating Schwartz Diamond 45°) structures are widely studied in literature due to their superior and multifunctional properties, enabling their integration in many physical applications [23]. The level-set approximation equations for the aforementioned TPMS-based structures are well listed in [23,26], and their isosurfaces can be seen in Figure 7a. Besides the traditional smooth and continuous topology of TPMS structures, Viswanath et al. [42] proposed a methodology to design a group of strut-based lattice structures derived from the isosurfaces of TPMS structures with greater fatigue performance as compared to the conventional solid elements.

#### 2.4.1. Categories of TPMS-Based Lattices and Design Strategies

TPMS-based lattices are classified into two types: sheet-based and ligament/solid/skeletal-based surface structures. The sheet and skeletal types of TPMS lattices, shown in Figure 7a,b, can be used for a variety of applications, including bio-scaffolding [43], thermal management applications [44], fluid flow enhancement [45], lightweight structural bending [26], among many other applications. As described in Figure 7a using the level-set approximation function, $\varphi_{G}$, 50–50% sheet-based ($\varphi_{sh}$) and ligament-based ($\varphi_{s}$) TPMS lattices are constructed by implicitly implementing the constraints: $\varphi_{sh}=-(\varphi_{G}^{2}-t^{2})$ and $\varphi_{s}=(\varphi_{G}-t)$, respectively, where *t* is an arbitrary constant that controls the thickness (or relative density) of the lattice. Constitutive mathematical relations that can predict the relative density of aforementioned types of TPMS-based architectures, as well as the link between the level-set approximation constant *c* and surface area-to-volume ratio (or surface-area density) can be found in Figure 6a. From Figure 6a, increasing the value of *t* increases and decreases the relative density of the sheet-based and ligament-based TPMS lattices, respectively, since they are a subtractive representation of each other from a solid-domain. Meanwhile, changing the value of *c* leads to variations in the topology/shape of the TPMS, leading in turn to unlimited number of shapes that can be obtained when assigning an infinite number of real values to *c.*

Designing intricate topologies for additive manufacturing, such as TPMS, is critical to its acceptance in engineering applications. Figure 7b depicts 3D printed polymer/polymer interpenetrating phase composite structures made of sheet-based or ligament-based TPMS lattices and solid material, with VeroWhite (a type of thermoplastic polymer) as the printing base material. Based on the 3D printed samples shown in Figure 7b, it is possible to comprehend this intricate class of lattice structures.

#### 2.4.2. Design Strategies for Grading TPMS-Lattice Topologies

**Figure 8:**
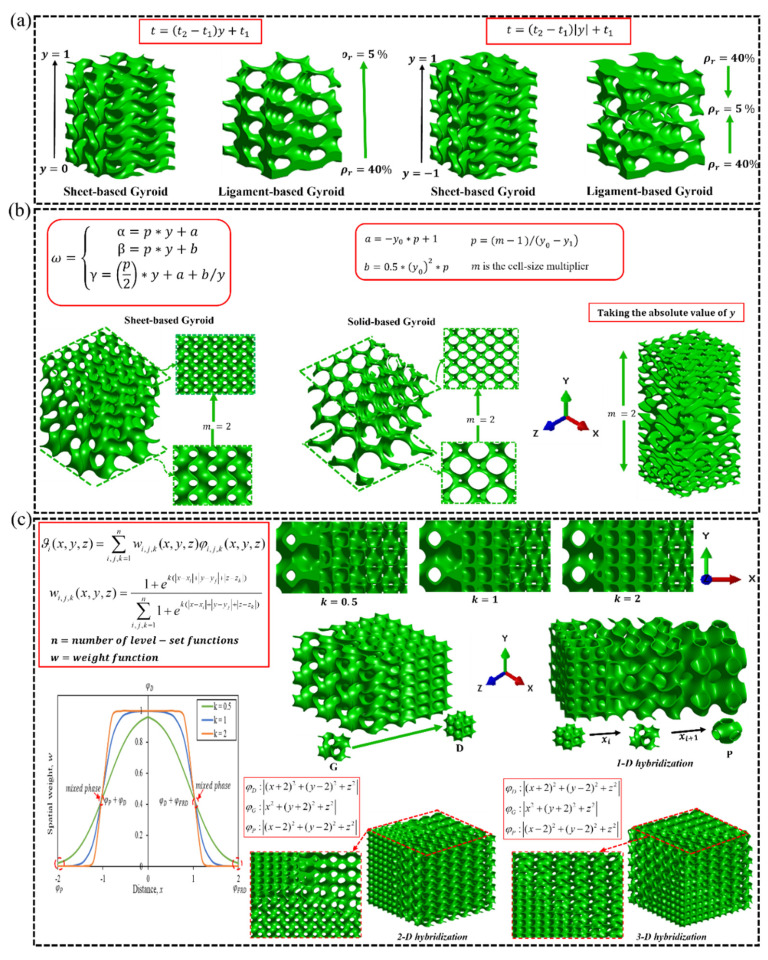
Design strategies for grading (**a**) relative density, (**b**) cell size and (**c**) topology of TPMS-based lattices.

Relative density, unit cell size and shape of lattices are the most important topological properties that are often graded/tuned to meet a specific engineering application. The relative density gradation of TPMS-based lattices is conducted implicitly based on a mathematical equation that expresses the parameter *t* as a function of the direction of gradation. Figure 8a shows a sheet-based and ligament-based Gyroidal lattice structure whose relative density is graded linearly and bi-linearly based on the provided expressions of the parameter *t*, where *t*_1_ and *t*_2_ are constants that determine the relative density of the G lattice at the start and end of gradation, assuming that $0\leq y\leq y_{max}$, respectively, where *y* is the axis of gradation. More complicated forms of lattice-topology relative density gradation can be achieved by expressing *t* as a specific function with single or multiple independent variables. On the other hand, the cell size of TPMS-based lattices can be graded following the implementation of the mathematical expression shown in Figure 8b, where α, β and γ are the spatial periodicity terms, *a* and *b* are constants, and *m* is the cell size multiplier such that a value of 2 means the cell size is multiplied by two at the end of the gradation. For example, Figure 8b shows the sheet-based and solid-based G lattice with cell size graded through its thickness assuming *m* = 2. Also, the shape of TPMS-based lattices can be hybridized to enhance the overall lattice mechanical property. To hybridize TPMS-based topologies based on their individual level-set approximation equations, a special weight function, most popularly the sigmoid function which is presented in Figure 8c, is utilized to achieve a smooth transition between the mono-topologies. However, to properly implement this weight function in the hybridization process, the parameter *k* that controls the transition rate must be determined (see Figure 8c). The reason behind this is the sigmoidal weight function does not yield values 0 and 1 at the start and end of hybridization, respectively, which is needed to fully implement the level set approximation equation at both bounds of topology hybridization, as shown in Figure 8c with k = 0.5. Hence, there is a need for more robust and computationally less expensive mathematical laws for hybridizing TPMS-based topologies, implicitly. Figure 8a provides examples of hybrid sheet-based TPMS lattices formed from hybridizing two topologies (i.e., G and D) and multiple topologies (i.e., D, G and P) in a single direction. In such instances, the other spatial terms (e.g., *y* and *z*) must be zero. However, the TPMS-based topologies can be hybridized in multiple directions if: *x*, *y* and *z* are not zero, and the transition regions can be user-controlled as shown in Figure 8c. For more in-depth details on grading the relative density of TPMS-based lattices, the readers are encouraged to refer to [23,26,46].

### 2.5. Stochastic-Based Metamaterials 

**Figure 9:**
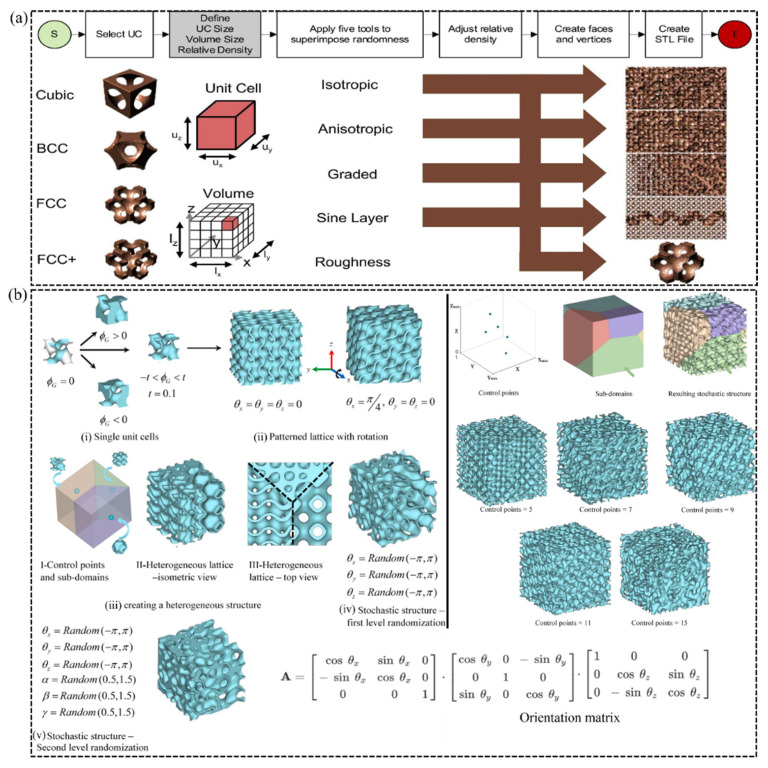
(**a**) The five techniques that can be implemented to the modeled volume: isotropic randomness, anisotropic randomness, graded randomness, layered randomness (Reproduced with permission from [47]. Copyright 2022, Elsevier). (**b**) The framework for generating stochastic sheet-based lattice materials; (i) design of single unit cells of Gyroidal structure, (ii) rotation of various patterned TPMS-based topologies, (iii) creating heterogeneous minimal surface stochastic lattice materials using control points and sub-domains, (iv) first-level randomization of heterogeneous structure via random orientation of Gyroidal sub-domains, (v) second-level randomization of heterogeneous structure via random orientation of Gyroidal sub-domains (Reproduced with permission from [24]. Copyright 2021, Elsevier).

A stochastic lattice is made up of randomly positioned points connected by beams or sheets within a volume or on top of surfaces. Several strategies for constructing stochastic-based materials have been discussed in the literature. For example, Groth et al. [47] proposed five simple tools which include isotropic randomness, anisotropic randomness, graded randomness, layered randomness, and surface roughness, as illustrated in Figure 9a. However, Al-Ketan et al. [24] developed a framework that facilitates the design of stochastic lattice materials from minimal surface topologies based on Gaussian-field randomness, as shown in Figure 9b. Currently, researchers have drawn much interest in utilizing minimal surface topologies in the latticing of engineering components since they offer better operational properties than strut-based lattice materials. Thus, it is of interest to discuss in detail how stochastic-based metamaterials are designed from minimal surface topologies. 

While Section 2.4 discussed the creation of regular and uniform lattices, Yang et al.’s [48] description of creating a heterogeneous lattice material composed of one or more TPMS types can still be followed. On this basis, the level-set equation is represented as the weighted sum of many level-set representations that are dispersed throughout space using control points to specify sub-domains in the way that [24]:

(2) $$\varphi_{Het}=\sum_{i=1}^{n}w_{i}(x)\varphi_{i}(x)$$

where $w_{i}(x)$ is the weight function as expressed in Figure 8c and n is the number of level-set approximation equations in the database. Every iso-surface in the sub-domains of the heterogeneous lattice material can be rotated locally using the 3 × 3 × 3 rotation matrix shown in Figure 9b, where θ is the rotation angle and is the control point at which rotation is performed [24].

### 2.6. Limitations 

Although several lattice architectures have been proposed in the literature with the sole purpose of meeting the desired engineering function, there are still aspects related to metamaterial designs that are yet to be explored to the best of the authors’ knowledge. Often, strut-based, plate-based and comb-based lattices are derived explicitly using CAD tools which is a time-consuming process. Thus, it will be interesting to be able to construct these classes of lattice materials implicitly to accelerate the designing process and facilitate functional grading of their topological properties.

## 3. Fabrication of Polymeric Metamaterials 

**Figure 10:**
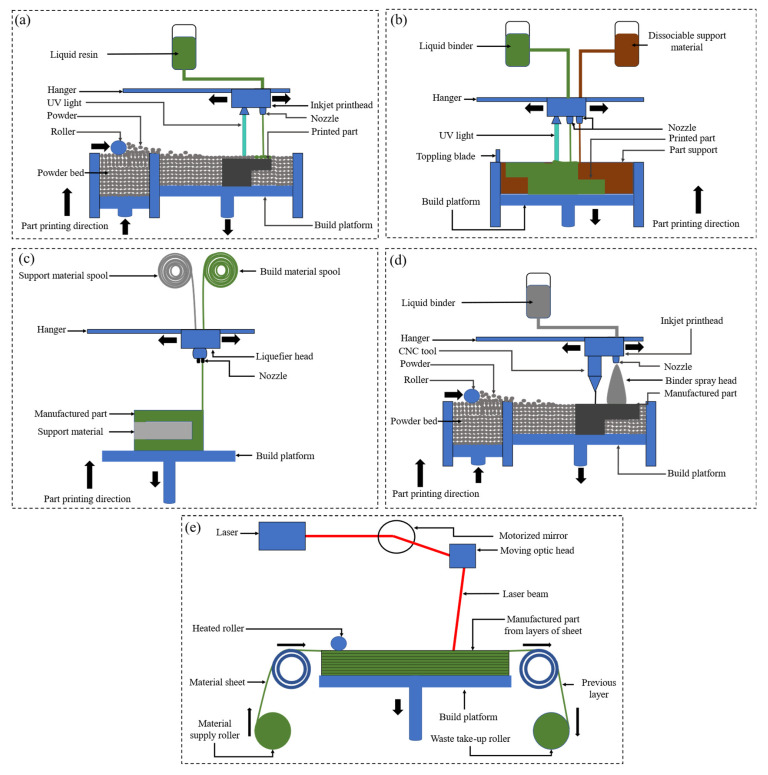
Illustration of (**a**) Vat photopolymerization, (**b**) Material jetting, (**c**) Material extrusion, (**d**) Binder jetting and (**e**) Sheet lamination polymer additive manufacturing processes.

Additive manufacturing (AM) is the commonly used fabrication technique to produce polymeric metamaterials. The base material could be supplied in many forms, such as photosensitive resins, filaments, viscous polymer inks, and thermoplastic powders. Different techniques of AM are utilized to produce polymeric metamaterials, where the most common techniques include binder jetting (BJ), sheet lamination (SL), vat photopolymerization (VP), material extrusion (ME), powder bed fusion (PBF) and material jetting (MJ) [49]. Each one of the aforementioned AM techniques is unique in terms of working principle and base material form [49,50]. Figure 10 describes the mechanism of the aforementioned AM techniques.

VP is a widely used AM process for manufacturing polymeric materials due to its well-known high resolution and part accuracy. As shown in Figure 10a, VP makes use of radiation (i.e., visible light and ultraviolet (UV) radiation) to construct the digital specimen through selectively polymerizing photosensitive liquid resins in a vat (i.e., liquid resin) [51,52]. The conception of stereolithography (SLA) VP technology has fueled the large-scale manufacturing of parts with higher resolutions [51,52]. 

Two-photon photopolymerization (2PP) is a direct laser printing technique which is another category of photopolymerization-based additive manufacturing technique known for its ability to print parts at a much higher resolution than SLA [49,53]. 2PP employs the two-photon absorption (TPA) concept, where two photons drive the electronic transitions rather than a single photon with visible or UV-based VP during the photopolymerization process [54]. TPA is derived from using a high photon-density laser beam, for example, a pulsed femtosecond laser. The part is built layer-wise and across the workspace by changing the focus of the laser in-line with the part’s geometrical configuration within the resin.

MJ is another polymer additive manufacturing technique whose process, as demonstrated in Figure 10b, is comparable to conventional 2D inkjet fabrication, where a liquid material is either deposited onto the workspace from the inkjet print-heads through a continuous process, or deposited-on-demand during manufacturing [49]. Afterward, the layer-wise deposited liquid material is solidified through photopolymerization. Due to the ability to use multiple printheads, the MJ technique is widely used for printing composites or multi-material components from a wide choice of materials such as resins, reactive materials, thermoplastics, wax, etc. [50]. Furthermore, using multiple material jets enables the manufacturing of functionally graded parts with smooth and high geometrical accuracy, thanks to the precise nature of the deposited material at the voxel level [55,56]. Recently, Polyjet and Multi-jet 3D printing (MJP) have been proposed by Stratasys and 3D Systems, respectively, which operate similarly to the conventional MJ process. However, what differentiates these processes is the type of support material used, for instance, water-soluble gel-like substances and paraffin wax are used as supporting materials in the Polyjet and MJP processes, respectively [49]. It is important to note that these support materials can be removed after the manufacturing process is completed through chemical dissolution or using high-pressure water for the Polyjet technique, and strictly by heat for the MJP technique [49]. 

Contrary to the above-discussed additive manufacturing techniques, ME, also referred to as fused filament fabrication (FFF) or fused deposition modeling (FDM), involves a progressive extrusion of viscous inks, polymer pellets or filaments through an orifice at elevated temperatures [57] onto the build platform as shown in Figure 10c, after which they solidify through cooling. FDM is the most commercially-viable additive manufacturing process, where thermoplastic filaments are used as feedstock. However, with the incorporation of extrusion-based systems in the recent development of the FDM process, pellets can be used as feedstocks which facilitate the processing of wider types of thermoplastics [58]. An example of the FDM process is direct ink writing (DIW) which also bears the name 3D plotting, 3D dispensing and 3D extrusion, which is known to print multiple materials.

BJ is a powder-based process that employs an inkjet printhead to selectively fuse the powder materials (e.g., metals, and ceramics, while polymers are mostly used as binders [59]) in a powder bed by dropping liquid binders while mimicking the shape of the 3D object as shown in Figure 10d. Due to the non-reliance of the BJ process on the heat source, much larger parts can be built through this process at a relatively low cost. Nevertheless, the absence of melting or sintering processes gives rise to relatively weak or flimsy and porous parts made by BJ, hence, the need for post-processing (typically through liquid infiltration and thermal sintering) of the fabricated part [49]. Unlike the BJ process, in the PBF technique, a powdered material is selectively fused using a heat source (e.g., laser or electron beam) on a powder bed. For example, the selective laser melting (SLM) method utilizes a laser source to completely fuse metal particles, thus, producing metallic components with higher mechanical resistance [22]. As the PBF technique involves recurring heating and cooling cycles during the solidification of deposited layers, the printed components experience the accumulation of unfavorable residual stresses. In this regard, Ahmed et al. [60,61] performed thermomechanical finite element analysis (FEA) to evaluate the thermal histories and residual stress evolution in the SLM process. It was found that the residual stresses lowered the effective elastic properties of the considered lattice materials with no effect on the plastic behavior of the material. 

With the SL process, 3D objects are printed by stacking and laminating feedstocks, which include papers, ceramic tapes, thermoplastic foils, and metallic and woven fiber composite sheets in the form of thin rolled sheets or foils. SL is a cost-effective process and can be used for fabricating relatively larger structures. The typical SL process is described in Figure 10e. Laminated object manufacturing (LOM) is one type of SL process that employs both subtractive and additive approaches to form 3D objects through the bonding of sheets. Inspired by this process, other cutting construction principles have been designed such as laser cutting, water jet cutting and computer numerical control (CNC) milling, and bonding types such as ultrasonic welding thermal bonding and adhesive bonding [49]. LOM is not widely used for commercial manufacturing due to issues related to the inception of internal cavities derived from the cutting process which contributes to unacceptable material waste [49].

### 3.1. Optimizing the Fabrication Procedure of Polymeric Metamaterials 

Geometrical imperfections and defects generated due to improper manufacturing deteriorate the mechanical performance of additively manufactured metamaterials [62]. The topic of enhancing the manufacturing fidelity of lattice materials has become of interest to several industries. For example, Holmes et al. [63] demonstrated the capability of Gyroid structures made of flexible thermoplastic polyurethane (TPU) to replace commercial soft padding foams to mitigate clinical conditions like pressure ulcers. Along the same lines, the manufacturing fidelity of lattice materials includes examining the deviations between the as-built (i.e., the printed) and the as-designed (i.e., CAD design) parts in terms of mass and geometry. Commonly, micro X-ray computed tomography (micro CT) is utilized to construct a 3D model of an as-built part to compare its manufacturing fidelity to an as-designed part. In fact, optimizing the process parameters is one of the key factors that improves manufacturing fidelity and mitigates the geometrical defects associated with developing lattice materials. Sala et al. [64] addressed the manufacturing complexities related to printing flexible honeycomb, Schwartz Primitive, and Gyroid lattices made of TPU using FDM. Certain process parameters were tuned to probe the optimum printing combination, such as printing temperature, retraction speed, retraction distance, printing speed, and fan speed. For example, in the case of a honeycomb with a unit cell size of 3 mm, the optimal process parameters included nozzle temperature of 230 °C, retraction distance of 1 mm, retraction speed of 2400 mm/min, printing speed of 1100 mm/min, and a build plate temperature of 50 °C.

Myers et al. [65] analyzed the effect of FDM process parameters (layer height, flow rate, and printing speed) on the printability and compressive strength of Schoen Gyroid and Schwartz Primitive structures made of PLA. A full factorial design was conducted to identify that there are three critical process parameters that influence the geometrical accuracy of a given print: layer height, flow rate and print speed, with four process parameters having a minor effect on the geometrical accuracy: nozzle temperature, build plate temperature, travel speed and retraction distance. It was found that the flow rate of material during printing (i.e., extrudability) was the most significant statistical parameter that influenced the geometrical accuracy of the test prints. Lower flow rate caused under extrusion (smaller geometrical dimensions) while higher flow rate caused over extrusion (larger geometrical dimensions). Furthermore, layer height was the next significant statistical parameter that affected the accuracy of the z-dimension (i.e., the height) and the strut diameter. Moreover, print speed had the smallest effect on the geometrical accuracy since it mainly controls the printing speed and layer adhesion. Aziz et al. [66] highlighted that surface roughness effects and the relative size of defects play a dominant role in determining the strength of lattice materials. It was suggested that the presence of manufacturing defects is likely to have a greater impact on the smaller size samples, given that the defects will occupy a larger percentage of the cross-sectional area. To this end, scaling effects were observed in the compression response of BCC lattice structures made of PLA, with the compression strength increasing by approximately 60% as the scale size was increased from 1/4th of the unit cell to the full length of the unit cell. It is important to highlight that one of the major challenges involved in the FDM technique is producing lattice materials that require minimal manufacturing and postprocessing time without compromising on print quality. Poddar et al. [67] argued that the traditional FDM process produces many weak polymeric interlayer bonds, for example, in the case of an octet-truss lattice material, vertical and diagonal trusses include dozens of weak interfaces between layers of small round or elliptical disks. To this end, the axial lattice extrusion process was introduced to improve the fabrication procedure of the conventional FDM process in which each vertical or diagonal truss is produced in one single continuous upwards extrusion motion rather than being printed as a lamination of multiple small area patches. It was reported that the octet-truss lattice made of fiber-reinforced ABS demonstrated near-perfect alignment of the chopped carbon fibers along the axes of the constituent trusses. 

Although the FDM process is most commonly utilized to fabricate polymeric composites, the PBF process produces parts with lower geometrical defects and higher machining accuracy when compared to the former [68,69]. As described by Gao et al. [70] in a comprehensive review, the interlayer bonding in the FDM process is known to be weak. Yet, many authors reported that geometrical deviations and surface roughness exist between the as-build and the as-designed parts even when using PBF processes [71,72,73,74]. Wang et al. [75] investigated the printability of Gyroid, Diamond, and I-WP TPMS structures made of porous poly-ether-ether-ketone (PEEK) using PBF process. Although the process parameters were optimized, the surface roughness and the relative density deviations were unavoidable. 

Fabricating a lattice material consisting of closed cells, such as plate lattices, is another challenge when considering the PBF process. These structures require the creation of circular holes on their surfaces for extracting the trapped and unfused powder material. This results in creating stress concentrations and decreasing the elastic moduli of these structures [76,77]. As an alternative, the material extrusion technique has been implemented by many authors to produce polymeric closed-cell lattice materials since it does not require the extraction of unprocessed material [40,78,79,80,81]. Nevertheless, the aforementioned studies emphasized the importance of mitigating manufacturing defects of lattice materials to attain improved performances. It is believed that the recent advancements in designing lattice materials using machine learning tools and enhancing their fabrication procedure based on intelligent algorithms would be one of the powerful solutions to mitigate the defects of additively manufactured lattice materials. For example, Zhou and Tian [82] utilized a machine learning algorithm to automatically determine a suitable filling path (i.e., the trajectory of the printing tool) for each sub-domain of the slicing layer of a lattice material, where different kinds of filling paths were utilized to demonstrate the efficiency of the proposed algorithm. Besides, Abdulla et al. [83] demonstrated the capability of kernel ridge regression in predicting the relative density of 3D printed specimens fabricated by the SLM technique based on the process parameters.

### 3.2. Polymeric Composite Materials 

Synthesizing lattice metamaterials that are capable of performing multiple structural functionalities increased the interest in multifunctional materials that produce structures with conflicting mechanical properties, such as high ductility with improved mechanical strength. The present section introduces major techniques that are implemented to produce multi-functional lattice metamaterials, particularly, the utilization of composite materials and multi-material additive manufacturing techniques. In brief, a composite material is defined as the assembly of multiple materials composed of a matrix that is strengthened by a reinforcer [84]. 

#### 3.2.1. Fiber Reinforced Composites

Fiber-reinforced composites are utilized to synthesize materials with a high strength-to-weight ratio. Different types of fibers are utilized to reinforce a host matrix, such as carbon fibers, glass fibers, and aramid fibers. For a thorough review of the different types of fibers, the readers are encouraged to refer to Prashanth et al. [85]. Furthermore, fiber alignment is one of the factors controlling the strength of fiber-reinforced composites. Günaydın et al. [86] demonstrated the printability of hexagonal and re-entrant structures made of nylon matrix reinforced with carbon fiber and glass fiber. The reinforcement approach enhanced the re-entrant lattice’s material-specific energy absorption, compressive strength and modulus values over the single nylon structure of 60%, 104% and 201%, respectively. 

Besides optimizing the fiber orientation in a host matrix, enhancing their fabrication procedure improves the mechanical behavior of the considered structure. Wang et al. [87] introduced a fabrication procedure to manufacture triangular corrugated structures (TCSs) made of continuous carbon fiber (CCF) reinforced thermosetting epoxy (EP) composite. The single-stroke printing path ensured a strong connection between the corrugated core and the face sheets. Subsequently, the CCF/EP TCS samples outperformed the ones made of unreinforced nylon, short fiber-reinforced nylon, and CCF-reinforced nylon in terms of compressive strength, stiffness, and energy absorption. Chikkanna et al. [88] argued that the selection of geometrical parameters and printing parameters of re-entrant diamond metamaterial is of prime priority to avoid premature failure of printed structures. It was reported that reinforcing the ABS matrix with chopped carbon and glass fiber enhanced the specific strength and stiffness of the re-entrant diamond structure at the cost of lowering the energy absorption capability. 

**Figure 11:**
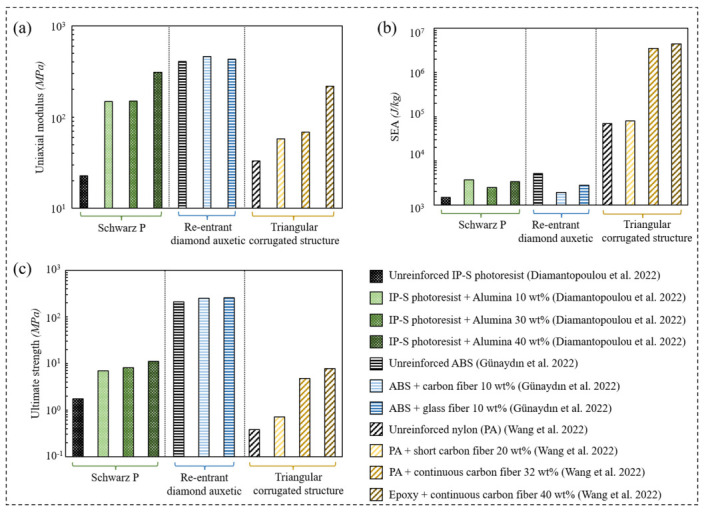
Bar charts demonstrating the mechanical properties of reinforced and unreinforced polymeric lattice materials: (**a**) Uniaxial modulus. (**b**) Specific energy absorption (SEA), and (**c**) Ultimate strength [86,87,89].

Figure 11 illustrates the mechanical properties of reinforced and unreinforced polymeric lattice materials in terms of uniaxial modulus, ultimate strength, and specific energy absorption (SEA). The data of Schwartz Primitive were taken from a study conducted by Diamantopoulou et al. [89], in which the sandwich construction of the Schwartz Primitive cell wall involved IP-S photoresist as the core material and alumina as the skin material. Figure 11a–c demonstrate that reinforcing the IP-S photoresist with variant weight percentages of the alumina enhanced the uniaxial compressive modulus, the SEA, and the ultimate strength of the considered structures, respectively. A similar trend is observed for the triangular corrugated structures [87]. However, the addition of carbon fiber and glass fiber with 10 wt% into the ABS matrix demonstrated an insignificant enhancement in the uniaxial modulus and the ultimate strength of the re-entrant diamond auxetic structures at the cost of SEA [83]. 

#### 3.2.2. Polymeric-Derived Ceramic Composite

**Figure 12:**
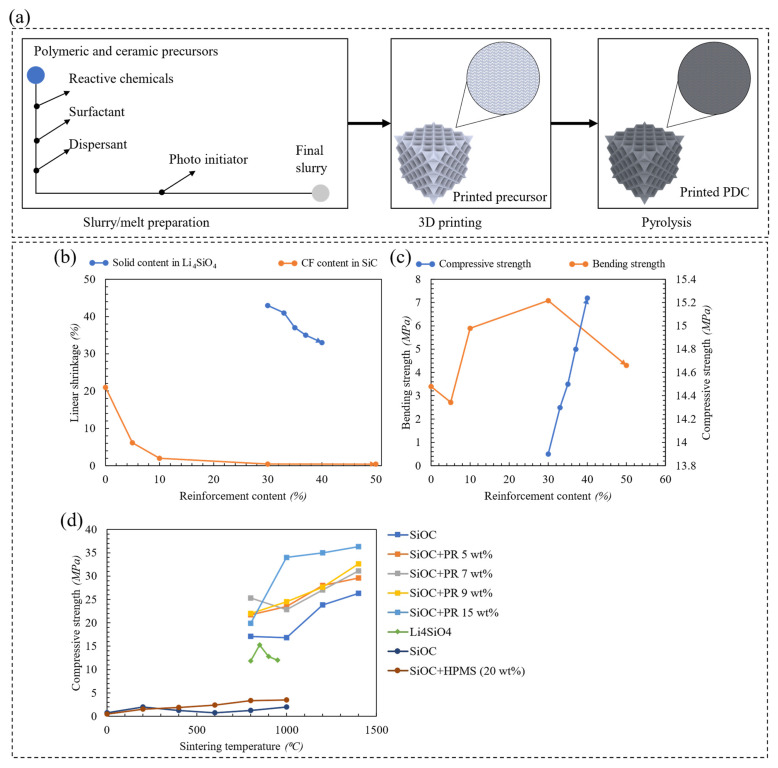
(**a**) General layout of producing an additively manufactured PDC lattice structure. (**b**) Demonstrating the effect of reinforcement content on the linear shrinkage of PDCs. (**c**) Demonstrating the effect of reinforcement content on the bending and compressive strengths of PDCs. (**d**) The effect of sintering/pyrolysis temperature on the compressive strength of PDCs.

Polymeric-derived ceramic (PDC) composite emerged as a potent material that exhibits high temperature resistant and excellent mechanical properties [90]. Figure 12a illustrates the general layout of producing an additively manufactured PDC lattice structure. Slurry preparation involves the mixing of polymeric and ceramic precursors with necessary chemicals to produce a precursor slurry or a precursor melt for laser/light-based [91] or extrusion-based [92] additive manufacturing techniques, respectively. When prepared for laser/light-based additive manufacturing, surfactant and dispersant are used to enhance the dispersity of ceramic particles into a polymeric resin. A photoinitiator is implemented to trigger the photopolymerization of the slurry mixture when exposed to ultraviolet light during 3D printing. After multiple heat treatment procedures, the printed precursor is pyrolyzed at high temperatures into a ceramic matrix to fill the voids in the printed precursor in a procedure known as polymer impregnation and pyrolysis (PIP). This procedure is repeated until a dense polymeric-derived ceramic composite is obtained [93,94].

Preparing a PDC composite for material extrusion technique, such as the DIW procedure, requires a material with efficient rheology (filament flowability). In DIW, the process material is provided as ink/paste composing of a polymeric material loaded with ceramic particulates. Very similar to the FDM procedure, an extrusion nozzle is utilized to deposit the ink under a controlled flow rate [95]. Liu et al. [91] elaborated on the rheological behavior and the linear shrinkage of carbon fiber-reinforced SiC composites produced via the DIW process. Linear shrinkage quantifies the decrease in fiber length when undergoing certain types of heat treatment. It was reported that increasing the content of carbon fiber decreased the viscosity of the printing inks which resulted in an excellent shear-thinning behavior and improved the extrudability of the ink for the DIW process. As depicted in Figure 12b, increasing the content of carbon fiber (CF) up to 30 wt% decreased the linear shrinkage of the investigated structure down to 0.5%. Not to mention that a lower linear shrinkage indicates a lower geometrical deviation between the designed and actual parts. Figure 12c demonstrates that the bending strength of the CF/SiC composites firstly decreased, then increased, and then decreased again with the increase in CF content. The initial drop in the bending strength is attributed to crack formation caused by the different shrinkage rates of CF and the SiC matrix that caused a weakening of the structure. However, in the case of the CF/SiC composite with a CF content of 10–30 wt%, the number of fibers exposed on single cracks increased and these fibers withstood the bending stress and the reinforcement effect of the fibers became dominant and strengthened the considered structure. Nevertheless, too many cracks were interconnected in the case of CF/SiC composite with a CF content of 50 wt% which deteriorated the performance of the composite and caused fracture. Clarkson et al. [96] argued that only a limited number of commercial pre-ceramic polymers meet the rheological specifications required by a conventional DIW system. Inks must be capable of flowing under shear through an extrusion nozzle and exhibit sufficient yield strength to be self-supported once extruded. To this end, the ultraviolet-assisted DIW (UV-DIW) was utilized which enables the usage of non-self-supporting inks with deficient rheology. A new type of UV-DIW ink was produced consisting of silicon carbide/silicon nitride composites that was promoted to be potent for aerospace applications. Moreover, polymers with high ceramic yield are too brittle to additively manufacture a lattice material through the FDM process. Zhao et al. [92] utilized a small amount of a thermal-plastic polymer (polypropylene with ≤5 wt%) to enhance the formability of a preceramic polymer (polycarbosilane). 

Digital light processing (DLP) technique is a vat photopolymerization additive manufacturing process that is commonly utilized to fabricate lattice materials made of photosensitive ceramic slurry. DLP operates under similar principles as the SLA procedure; however, a UV-light source and optical systems are utilized instead of a laser beam to cure a prepared slurry in the DLP procedure [97]. Su et al. [98] proposed a fabrication procedure in which low-cost and environmentally insensitive precursors are utilized to produce Li_4_SiO_4_ powder with enhanced microstructural stability and higher phase purity when compared to the existing Li_4_SiO_4_ PDC. Figure 12b demonstrates that an increase in solid content reduced the resin content within the system which resulted in a decrease in linear shrinkage. Furthermore, as the solid content increased, the micro-pores between grains closed resulting in an increase in the sample’s density. As depicted in Figure 12c, this is also reflected as an increase in the compressive strength of the considered sample. Moreover, Figure 12d demonstrates the compressive strength of the samples after sintering at various sintering temperatures. It turned out that sintering the samples beyond 800 °C increased the porosity of the samples and alleviated their compressive strengths. Furthermore, Figure 12d summarizes the outcomes from Xiong et al. [99] and He et al. [100] that indicate increasing the solid content/reinforcement in a host matrix at fixed sintering temperatures, increases the compressive strength of PDCs.

Investigating the effects of heat treatment and linear shrinkage on the strength of PDCs were extensively exploited in the current state of the art. However, impact absorption of PDCs under extreme temperatures should also be explored to promote the applicability of these materials for impact absorption under extreme heat conditions, such as the ones found in space system applications. 

#### 3.2.3. Cementitious Composite

Concrete has been widely implemented in the construction industry due to its high compressive strength capability. However, owing to its brittleness and low tensile strength, reinforcing the cementitious composite with metals and polymers emerged as a viable solution to enhance their ductility [101]. Most commonly, steel rebars are used as reinforcement elements to improve the ductility of cementitious materials at the cost of corrosion problems [102]. As an alternative, 3D-printed polymeric lattices are utilized as reinforcement structures to enhance the ductility and improve the crack resistance of concrete without initiating corrosive reactions. Salazar et al. [103] enhanced the ductility of ultra-high-performance concrete (UHPC) by reinforcing it with octet-truss lattices made of PLA and ABS materials. The polymeric lattices were placed into beam-shaped molds and then infiltrated with UHPC to form the lattice-reinforced concrete beams. It turned out that strengthening the UHPC with a 33.7% PLA reinforcing ratio enhanced the peak load and the toughness by 54.6% and 8650%, respectively. The addition of PLA into the UHPC cementitious composite completely shifted the failure mode from brittle to ductile.

**Figure 13:**
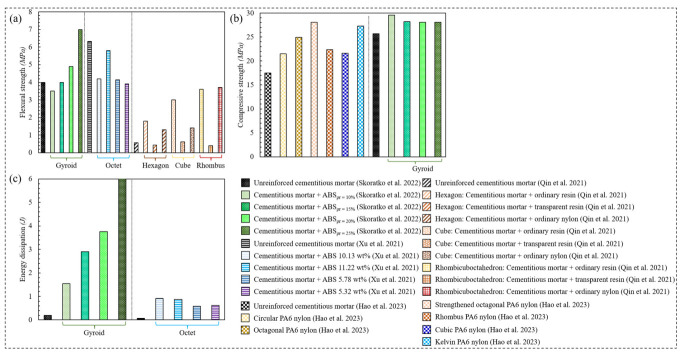
Bar charts demonstrating the mechanical properties of reinforced and unreinforced cementitious composites: (**a**) Flexural strength. (**b**) Compressive strength, and (**c**) Energy dissipation [101,102,104,105].

Qin et al. [104] investigated the influence of altering the base material (ordinary resin, transparent resin, nylon) and the geometry (hexagon, cube, rhombicosidodecahedron/rhombus) of 3D printed polymeric lattices on the flexural strength of cementitious backfill composites reinforced with the latter polymers. Figure 13a demonstrates that incorporating the polymeric lattice materials into the cementitious composite always increased its flexural strength, except in the case of reinforcing the composite with a rhombus lattice material made of transparent resin. It turned out that the rhombus lattice made of ordinary nylon demonstrated the largest improvement in the flexural strength of the cementitious composite, followed by the rhombus, the cube, and the hexagon lattices made of ordinary resin.

Xu et al. [102] argued that a relatively high reinforcing ratio is required to reinforce a cementitious composite with conventional polymeric metamaterials. Instead, the utilization of functionally graded polymeric structures enhances the ductility of the cementitious composite with a much lower reinforcing ratio. Octet lattice materials were functionally graded to optimize the flexural performance of the cementitious composite with variant ABS reinforcing ratios. Figure 13c demonstrates that reinforcing the cementitious composite with 5–11% ABS reinforcing rations greatly enhanced the energy dissipation at the cost of lowering the flexural strength of the cementitious composite. 

Besides improving the ductility and flexural strength of cements, Hao et al. [105] enhanced the compressive properties of 3D printed polyamide 6 (PA6) lattice-reinforced cementitious composites with six different designs, particularly: circular, octagonal, strengthened octagonal, rhombus, cubic, and kelvin lattice materials. As depicted in Figure 13b, the strengthened octagonal enhanced the compressive strength of the cementitious composite by 60.7%, followed by the kelvin, the octagonal, the rhombus, the cubic, and the circular lattice materials, respectively. Along the same lines, Skoratko et al. [101] harnessed the Gyroid lattice material as the reinforcing element to enhance the ductility and strength of cementitious mortar beams. The Gyroid reinforcers were made of ABS with four different relative densities, 10%, 15%, 20%, and 25%. It was reported that the properties of the Gyroid-reinforced cementitious composites were highly quasi-plastic and comparable to those of steel fiber-reinforced cementitious composites. As depicted in Figure 13a, the cementitious composite reinforced with ABS of 10% relative density exhibited a flexural strength which was 10% lower than that of the unreinforced cement matrix. However, gradually increasing the relative density of the ABS plastic up to 25% enhanced the flexural strength of the cement matrix by 75%. In terms of compressive strength, the cementitious composite reinforced with ABS of 10% relative density exhibited the highest performance, where increasing the ABS relative density above this point decreased the performance of the reinforced cementitious mortar. Remarkably, Figure 13c demonstrates that reinforcing the cementitious mortar with ABS of 25% relative density improved its energy dissipation by 2910%. 

The aforementioned studies indicate the increasing interest in utilizing 3D-printed polymeric lattice materials as spatial reinforcing elements to enhance the ductility and strength of cementitious composites. It was highlighted that the enhancement of certain types of mechanical properties is attained at the cost of worsening some other performance markers. To this end, it is believed that the utilization of multi-objective optimization tools would support the enhancement of conflicting mechanical properties without deteriorating a specific property at the cost of another. Furthermore, since the performance of polymeric lattice materials is sensitive to temperatures, the current state of the art seems to lack explanations for the effect of thermal gradient on the mechanical performance of cementitious composites reinforced with polymeric lattice materials. 

### 3.3. Multi-Material Additive Manufacturing 

**Figure 14:**
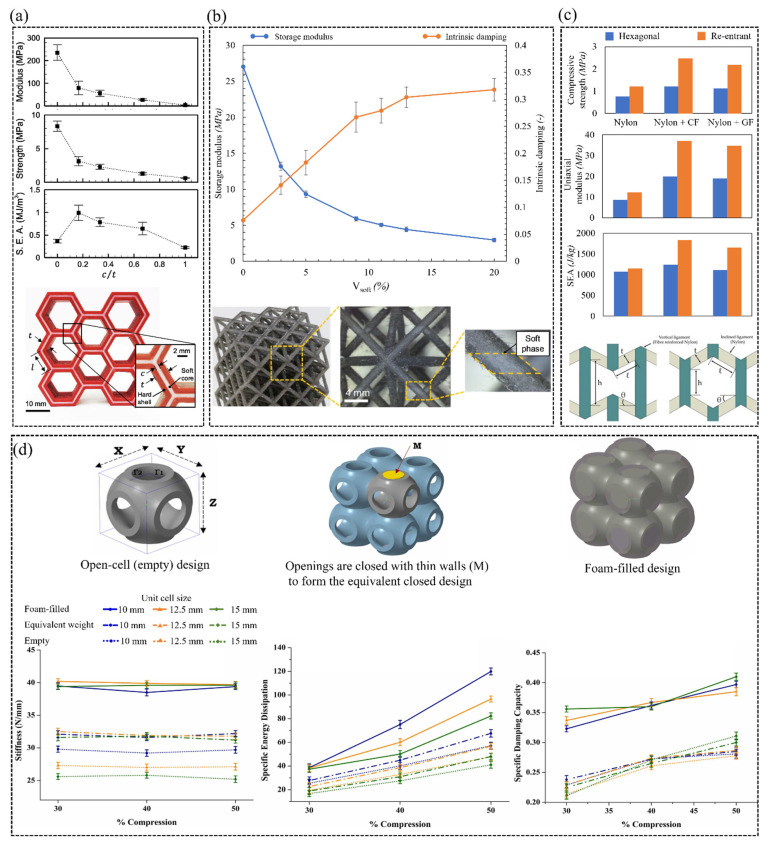
Illustrations of design and performance of multi-material polymeric lattice materials. (**a**) Demonstration of the design and mechanical properties of honeycomb structures made of PLA and TPU (Reproduced with permission from [109]. Copyright 2022, Elsevier). (**b**) Demonstration of the effect of varying the volume fraction of the soft phase embedded into the stiff CFRP members on its storage modulus and intrinsic damping (Reproduced with permission from [106]. Copyright 2020, Elsevier). (**c**) Demonstration of the improvement in compressive strength, uniaxial modulus, and SEA of re-entrant and hexagonal lattice materials due to embedding glass/fiber reinforcement into nylon structures (Reproduced with permission from [86]. Copyright 2022, Elsevier). (**d**) Demon-stration of the designs and the mechanical properties of multi-material SU lattice materials (Re-produced with permission from [110]. Copyright 2022, Elsevier).

Despite the distinguished mechanical performance of metamaterials, achieving a combination of conflicting properties is challenging with single material composition [106]. For example, ceramic-based metamaterials are superior in terms of compressive strength but inferior in terms of energy dissipation due to their brittle nature [107,108]. To this end, multi-material additive manufacturing is exploited to produce structures characterized by conflicting properties, such as attaining efficient stiffness and energy absorption capabilities. For example, Yavas et al. [109] fabricated honeycomb structures composed of variant ratios of hard and soft materials, in which the hard shell was made of PLA to maintain stiffness, and the soft core was made of TPU to enhance energy absorption. The considered structures were fabricated using a multi-material FDM printer equipped with a dual extrusion system such that each nozzle deposits one type of material. The study explored the effect of varying the TPU:PLA ratios (i.e., c/t ratios depicted in Figure 14a) on the uniaxial modulus, the compressive strength and the energy absorption of the considered structures. It was reported that increasing the c/t ratio from 0 to 1 transformed the failure modes from brittle to ductile. 

Xu et al. [106] argued that carbon fiber reinforced polymer (CFRP) composite is superior in terms of stiffness-to-weight ratio but inferior in terms of energy dissipation. The study utilized a multi-material projection micro-stereolithography process to fabricate octet-truss lattice material comprising of a fraction of soft phase material embedded within the stiff CFRP strut members. Figure 14b illustrates that by varying the volume fraction of two constituent materials, the intrinsic damping of the stiff CFRP was astonishingly improved at the cost of weakening the storage modulus. Along the same lines, K. Günaydın et al. [86] embedded two types of reinforcements (glass fiber (GF) and carbon fiber (CF)) into the vertical ligaments of re-entrant and hexagonal lattice materials made of nylon. Figure 14c demonstrates that the multi-material approach enhanced the re-entrant lattice material’s SEA, compressive strength and modulus values over the single nylon structure of 60%, 104% and 201%, respectively. Furthermore, an improvement of 15%, 60% and 127% in the same mechanical properties was observed for hexagonal lattice material.

Prajapati et al. [110] introduced a hybrid 3D printing and foaming process based on FDM technique which involved simultaneous printing and foam-filling of a biomorphic sea urchin (SU) lattice material using TPU as the primary material and polyurethane (PU) foam as the secondary filling material, respectively. The filled lattice materials exhibited higher stiffness, energy dissipation and damping characteristics than those of the unfilled designs. Figure 14d demonstrates the designs and the mechanical performance of the considered structures. Chen and Zheng [111] introduced an additive manufacturing platform based on the DLP technique in which re-entrant lattice materials were fabricated using two constituent materials with large stiffness gradients composed of ethoxylated bisphenol A-dimethacrylates (material A) and ethoxylated trimethylolproane-triacrylate (material B). The system was equipped with a self-cleansing robotic dispenser that allowed simultaneous switching between materials and cleaning residue monomer at each sequential layer before a new feedstock material was perfused. The compressive modulus and Poisson’s ratio of the considered structures were investigated at various material B: material A ratios (B:A), where an increase in compressive modulus was reported at higher ratios of B:A. Furthermore, in contrast to conventional auxetic metamaterials whose negative Poisson’s ratio is dictated by their geometry, multi-material auxetic metamaterials can display Poisson’s ratios from extreme negative to zero, independent of their 3D architecture. The authors stated that simultaneous tuning of Poisson’s ratio and moduli within the 3D multi-materials could open up a broad array of material-by-design applications ranging from flexible armor and artificial muscles to actuators and bio-mimetic materials. One of the recent implementations of multi-material additive manufacturing is the utilization of self-healing polymers in biomedical devices and robotic applications. Self-healing polymers are capable of self-repair from physical damage by utilizing reversible interactions between the molecules [112]. However, self-healing polymers are usually synthesized at the cost of mechanical strength. Huang et al. [113] fabricated three types of architects (honeycomb, re-entrant, and chiral) made of a multi-material self-healing polymer composed of hard (4-acryloylmorpholine) and soft thermoplastic (monofunctional urethane) composite. Interestingly, a healing efficacy of 80% enabled the printed structures to recover their structural integrity and stiffness after fracture. 

Multi-material printing produces multifunctional lattice metamaterials with conflicting mechanical properties using the same geometrical design. It is believed that partitioning a design into hard and soft regions would offer a route towards utilization of multifunctional architected structures in areas that require efficient load bearing and mass transport capabilities, such as thermofluidic and tissue engineering applications [114].

### 3.4. Polymeric Metamaterials’ Fabrication Challenges

Regardless of the fabrication technique, each kind of additive manufacturing process imposes challenges on synthesizing defect-free polymeric structures. Table 2 summarizes a few of the fabrication solutions for common manufacturing challenges encountered while producing polymeric metamaterials. For example, He et al. [100] and Essmeister et al. [115] introduced silica-based particulates to overcome strut deformation and crack propagation while manufacturing PDC composites, respectively. Furthermore, Chen and Zheng [111] mitigated the cross-contamination between different material feedstocks during multi-material additive manufacturing by integrating a self-cleansing robotic dispenser into a micro-SLA 3D printer for cleaning residue monomer at each layer. Further examples are reported in Table 2. 

## 4. Mechanical Characterization of Polymeric Metamaterials 

In terms of polymeric metamaterials, various mechanical tests have been attempted to investigate their mechanical properties. The most common mechanical test for polymeric metamaterials, and in fact for all metamaterials, is the quasi-static uniaxial compression test. In addition, other tests have been attempted for polymeric metamaterials, including quasi-static bending tests and dynamic impact tests. In the following sections, each of these different mechanical tests are discussed and the performances of various polymeric metamaterials are reviewed.

### 4.1. Uniaxial Compression Tests

One of the simplest mechanical tests that exist is the uniaxial compression test, where a specimen is used to conduct the test and investigate the compressive behaviors of polymeric metamaterials. Various mechanical properties can be obtained from uniaxial compression tests, such as the elastic modulus, yield strength and ultimate strength. 

**Figure 15:**
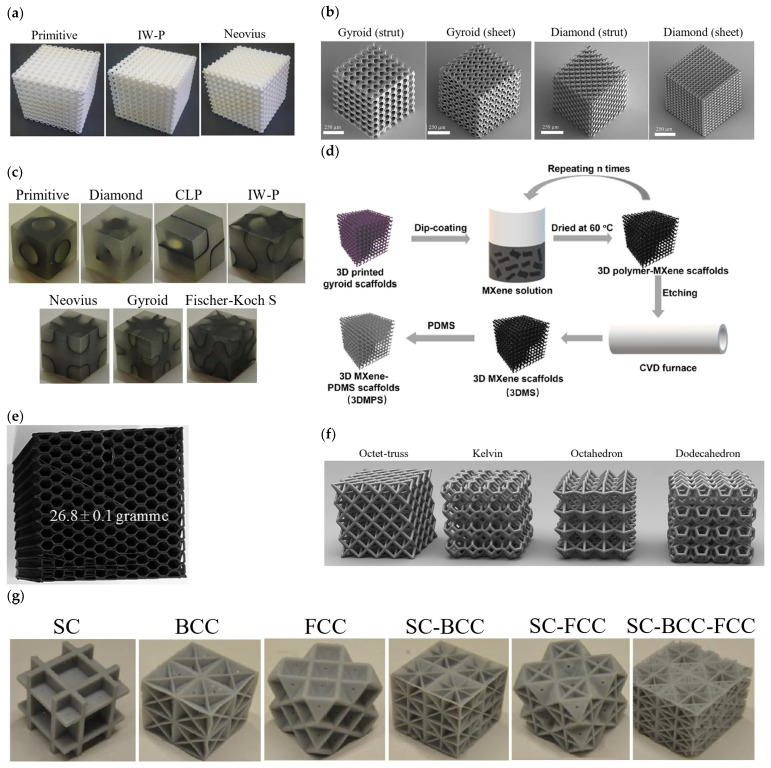
Various polymeric specimens used for mechanical testing. (**a**) Sheet-based TPMS cubic specimens (Reproduced with permission from [119]. Copyright 2017, Elsevier). (**b**) Strut- and sheet-based TPMS cubic specimens (Reproduced with permission from [120]. Copyright 2018, Wiley). (**c**) Sheet-based TPMS interpenetrating phase composite specimens (Reproduced with permission from [118]. Copyright 2016, Elsevier). (**d**) The steps of manufacturing the 3D MXene scaffold with polymer-based, strut-based Gyroidal TPMS [123]. (**e**) Hexagonal honeycomb structure (Reproduced with permission from [130]. Copyright 2018, Elsevier). (**f**) Strut-based cubic specimens (Reproduced with permission from [131]. Copyright 2021, Wiley). (**g**) Uniform and hybrid strut-based cubic specimens (Reproduced with permission from [79]. Copyright 2021, Elsevier).

Due to their superior properties, many studies have investigated the mechanical properties of TPMS lattices under uniaxial compression tests [117,118,119,120,121,122,123]. Uniform TPMS lattices have been investigated by Abueidda et al. [119], specifically sheet-based TPMS cubic samples of Schwartz Primitive, Schoen’s I-WP and Neovius (Figure 15a). The cubic samples were printed using the SLS technology with PA12 material, where different specimen sizes were investigated with a unit cell edge length of 1.5 cm each. The elastic modulus and the ultimate strength of these three sheet-based TPMS lattices were investigated for a relative density range of 5% to 26%. It was found that the I-WP and Neovius structures had higher uniaxial compressive moduli and uniaxial strengths than the Primitive lattice. In addition, the study conducted numerical simulations on these structures using finite element method and has found reasonable agreement with experiments. It is worth noting that the bulk material was modeled using two different constitutive models (Arruda-Boyce [124,125] and flow evolution network [126]), where both have shown very similar results to each other. It should be noted that a proper constitutive model calibration improves the capability of numerical models in predicting the large deformation behavior of polymeric lattice materials, particularly the ones fabricated with base materials that are highly sensitive to strain and temperature variations. With this regard, Almomani et al. [127] and Shahin et al. [128] provided constitutive model calibrations of the time and temperature-dependent behavior of HDPE material.

**Figure 16:**
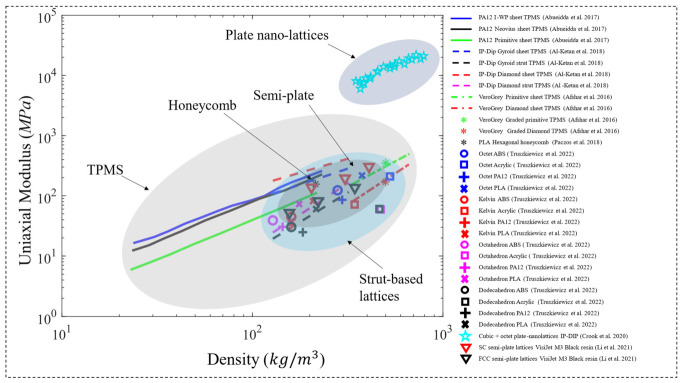
Uniaxial modulus versus metamaterial density from uniaxial compression physical tests on various polymeric metamaterials [76,77,117,119,120,126,131].

**Figure 17:**
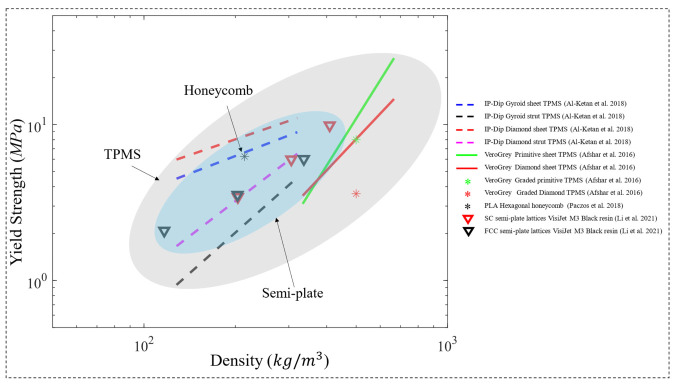
Yield strength versus metamaterial density from uniaxial compression physical tests on various polymeric metamaterials [76,117,120,130].

In a subsequent study, Abueidda et al. [121] investigated the Gyroid sheet-based TPMS lattice in a similar manner and found that the uniaxial compressive properties of the Gyroid rank between the Neovius (highest properties) and the I-WP lattices. Similarly, another study [120] investigated uniform TPMS lattices. However, both sheet-based and strut-based TPMS lattices were investigated in this study, based on the Diamond and Gyroid topologies (Figure 15b). In addition, the strut-based Octet-truss lattice was investigated in [120] to compare and contrast with the TPMS lattices’ performance. 100 unit cells with tessellated cubic samples, manufactured using the dip-in laser lithography configuration, were tested under uniaxial compression loading. The properties investigated were the uniaxial modulus and yield strength, for a relative density range of 10% to 25%. The study has concluded that when compared to the Octet-truss and TPMS strut-based micro-lattices, TPMS sheet-based micro-lattices display superior mechanical properties. In terms of the uniaxial modulus (Figure 16) and yield strength (Figure 17), the ranking from highest to lowest performing lattices is Diamond sheet-based TPMS, Gyroid sheet-based TPMS, Diamond strut-based TPMS and finally the Gyroid strut-based TPMS, respectively. Al-Ketan et al. [120] also investigated the mechanical properties of these structures through finite element simulations using only an elastic-perfectly plastic material model. Generally, it is found that the finite element simulations predict higher stiffness values than the experiments, especially at lower relative densities. These variations can be attributed to printing defects and/or limitations of the finite element material model used that does not account for buckling or damage.

Similarly, Afshar et al. [117] and Zhang et al. [122] investigated TPMS lattice cubes under uniaxial compression tests, but not only using uniform TPMS lattices. Afshar et al. [117] investigated both uniform lattices and linearly graded lattices in terms of relative density, for both Primitive and Diamond strut-based TPMS structures. The cubic specimens were made from VeroGrey FullCure850 photopolymer resin (Stratasys, Ltd., Rehovot, Israel). The properties investigated were the elastic modulus and yield strength, for a relative density of 30% and 60% in terms of the uniform lattices, and for a relative density variation of 30% to 60% for the linearly graded lattices. Results showed that the uniform lattices of 60% relative density had the highest elastic modulus and yield strength for both structures (Primitive and Diamond), which is an expected outcome, where the Primitive structure showed much higher values than the Diamond structure (Figure 16 and Figure 17). On the other hand, Zhang et al. [122] investigated the elastic moduli and yield strengths of different groups of specimens, which are uniform TPMS lattices and functionally graded lattices in terms of relative density, lattice topology and both. The study focused on strut-based TPMS structures made from photopolymer resin, which are Schwartz Primitive, Schoen’s I-WP, Gyroid and Diamond. In terms of the uniform TPMS group and functionally graded lattices in terms of the relative density, the specimens’ relative densities ranged between 40 and 60%. In terms of the functionally graded lattices (topology-wise), a combination of three topologies is used in each specimen, for example, I-WP with Gyroid and Diamond at a constant relative density or varying relative densities. Generally, results showed that uniform I-WP lattice showed the highest elastic moduli and yield strength between all topologies and functionally graded lattices considered.

On the other hand, Dalaq et al. [118] conducted uniaxial compression tests on polymeric TPMS-based interpenetrating phase composites (IPCs) using PolyJet 3D printers. The cubic samples were made of Tango-Plus (FLX930, Stratasys, Ltd) [129], a thermoplastic elastomer with rubber-like properties, while the reinforced TPMS structure is made of Vero-Plus (RGD875, Stratasys, Ltd) [129], a strong thermoplastic. The TPMS sheet lattices considered were Schwartz Primitive, Diamond-rhombic, Schwartz CLP, Schoen I-WP, Neovius, Gyroid and Fischer-Koch S (Figure 15c), where all the specimens were reinforced with only one unit cell of each TPMS lattice at a fixed relative density. Dalaq et al. [118] investigated the compressive elastic modulus and ultimate strength of these IPCs. In terms of enhancement of the mechanical properties of the matrix, the elastic modulus significantly increased with all architectures. However, in terms of the matrix strength, only the Primitive lattice managed to enhance the strength of the matrix. In terms of the values, CLP-IPC showed the highest elastic modulus, while the Primitive-IPC showed the highest ultimate strength. Lately, in an attempt to manufacture a novel piezoresistive sensor, Fu et al. [123] investigated the compressive elastic modulus and ultimate strength of a 3D MXene scaffold with polymer-based, strut-based Gyroidal TPMS as the initial sacrificial scaffold (Figure 15d). The cubic samples consisted of unit cells in three dimensions with a total specimen length of 2 cm, at a relative density of 15%. 

**Figure 18:**
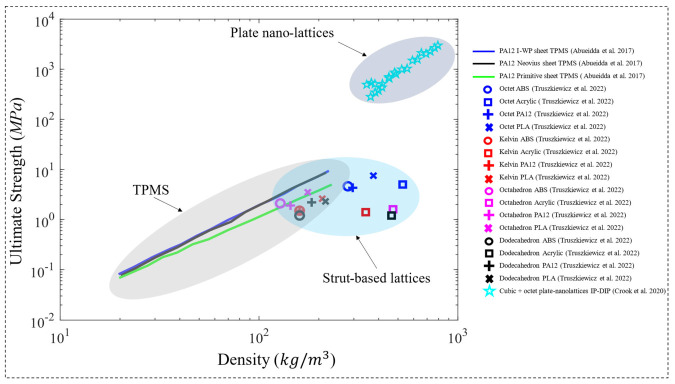
Ultimate strength versus metamaterial density from uniaxial compression physical tests on various polymeric metamaterials [77,119,131].

Other than TPMS structures, other polymeric metamaterials have been investigated as well. Paczos et al. [130] investigated the compressive elastic modulus and yield strength of polymeric hexagonal honeycomb structures (Figure 15e). In an attempt to investigate the properties of various short sandwich beams, the properties of a cubic specimen made of uniform honeycomb lattices using PLA were investigated and plotted in Figure 16 and Figure 17. In addition, the compressive properties of the Octet-truss, Kelvin, Octahedron and Dodecahedron lattice structures (Figure 15f) were investigated by Truszkiewicz et al. [131]. Each of the structures was manufactured with four different polymeric materials using different printing techniques, as cubic specimens with a size of 3 mm. The materials considered are acrylic resin, acrylonitrile ABS, PLA and PA12. However, each structure is printed at one relative density that is different for each structure. Results indicate that the octet-truss structure showed the highest compressive properties (elastic modulus and ultimate strength) between all structures, for all materials considered, as shown in Figure 16 and Figure 18. In addition, Truszkiewicz et al. [131] used finite element simulations to investigate the stress-strain response of these structures with various materials. An elastic-plastic material model was used to represent the bulk material properties, where the model is obtained from bending tests of solid specimens made from the materials used, by fitting the plastic section to a Voce extrapolation model. The results show a good agreement between the experiments and numerical simulations.

On the other hand, various studies [76,77,78,80] investigated the mechanical properties of plate lattices under uniaxial compression loading. The lattice structures explored by Ubaid et al. [80] were additively manufactured using multiwall carbon nanotube (MWCNT) incorporated polypropylene random (PPR) copolymer filaments. PPR/CNT composite cellular structures consisted of 2 × 2 × 2 unit cells with varying CNT content (0, 4 and 6%) and relative density (20 and 30%). The cellular structures were printed using three different architectures: BCC plate–lattice, Kelvin foam, and Gyroid–lattice. Compared to Kelvin foam and Gyroid lattice, the BCC plate-lattice showed much higher elastic modulus values. Even though the modulus values were reported, the mass and density of each specimen (with the specific CNT content) were not reported clearly. Crook et al. [77] investigated the elastic modulus and compressive strength of polymeric plate-nanolattices printed from photoresist IP-DIP using two-photon polymerization direct laser writing. The cubic + octet topology was used to represent the closed-cell architecture plate nanolattices with 5 × 5 × 5 unit cells at 25%–60% relative density. The results of the study indicated that these plate nanolattices have high stiffness and strength in compression compared to other existing cellular materials at the same relative density. Li et al. [76] investigated a range of semi-plate lattices printed from VisiJet M3 Black (3D Systems, Inc., York County, SC, USA) resin using a multi-jet printing 3D plastic printer. The study investigated the elastic modulus and yield strength of two semi-plate lattices (simple cubic and face center cubic symmetries) using cubic specimens that consisted of 4 × 4 × 4 unit cells with a unit cell size of 10 mm for a range of 20%–40% relative densities. Furthermore, Almesmari et al. [78] investigated the mechanical properties of BCC plate lattices made of ABS under uniaxial compression loading, at relative densities of 20%, 30%, and 40% with specimens having different combinations of unit cell lengths and plate thicknesses. The study investigated the number of unit cells needed to obtain the effective properties of the lattice and found that cubic samples consisting of 2 × 2 × 2 unit cells were sufficient. Results showed that the BCC plate lattice has stiffness values, that outperform other surface lattices. However, the density of the bulk material or the mass of the specimens was not reported.

All the results (uniaxial modulus, yield and ultimate strengths) reported by the previously discussed studies are plotted in terms of the densities of the metamaterials, whenever the density is either reported or can be calculated, as presented in Figure 16, Figure 17 and Figure 18. In terms of the uniaxial modulus (Figure 16), and for a specific density, the cubic + octet plate nano-lattices (IP-DIP) show extremely high values compared to all other topologies and materials considered. Following that, the PA12 sheet-based TPMS lattices have the highest moduli compared to the IP-DIP strut-based TPMS lattices and the other strut-based lattices (various materials) that have similar moduli. In terms of the yield and ultimate strengths, it is clear that there is a lack of density range of results and a variety of lattices reported in the literature. Thus, comparison between different classes is not possible. There is definitely a need for more studies on polymeric metamaterials with a wide range of densities and different metamaterial classes, such as plate lattices and honeycombs. This being said, it is important to highlight the high strength values of the cubic + octet plate nano-lattices (IP-DIP), shown in Figure 18.

It should be noted that polymeric metamaterials produced at the nano-scale are distinct from polymeric nanomaterials that are composed of natural and chemically synthesized polymer materials in the form of, nano-particles, -rods, and -tubes, produced via nanotechnology with at least one dimension between 1 and 100 nm [132]. Recently, there has been an increasing interest in developing vaccine delivery systems using polymeric nanomaterials as they feature high biological safety and good biodegradability [133]. For more in-depth details on the implementation of polymeric nanomaterials in wastewater treatment, forensic analysis, and phytochemicals, the readers are referred to the following references [134,135,136], respectively.

### 4.2. Bending Tests

The flexural properties of materials are important as well to characterize the performance of polymeric metamaterials, which can be obtained using three-point or four-point bending tests. Several studies have attempted these tests on polymeric metamaterials and reported their flexural properties. A couple of studies investigated the flexural properties of polymeric honeycomb structures [137,138]. Li and Wang [138] conducted three-point bending tests on composite sandwich structures made of acrylic-based photopolymer honeycomb as the core structure. Two different honeycomb structures were considered: the conventional honeycomb and the re-entrant honeycomb. In terms of the face sheets, three different face sheet materials were used to manufacture different specimens, which were acrylic-based photopolymer, woven carbon fiber reinforced polymer and unidirectional carbon fiber reinforced polymer. By investigating the flexural stiffness, flexural strength and the energy absorption of these specimens, it was found that the conventional honeycomb structures had higher values than the re-entrant honeycomb at a corresponding relative density. However, due to the relatively homogenous stress distribution, the re-entrant honeycomb sandwich constructions exhibited an interesting global failure mode. Additionally, the sequential snap-through instabilities in the re-entrant honeycomb sandwich structures considerably increased the energy absorption capacity. In contrast, because of the localized stress concentration, traditional honeycomb sandwich structures exhibited catastrophic failure earlier. The polymeric honeycomb sandwich structures have also been investigated under three-point bending by de Castro et al. [137]. PLA thermoplastic was used to manufacture the sandwich structures for three different core designs which are: hexagonal honeycombs oriented (i) out-of-plane and (ii) in-plane, and S-shape corrugated. With a similar mass for all three samples, the results showed that flexural modulus and strength were highest for the out-of-plane oriented honeycomb structure, followed by the S-shape corrugated and in-plane oriented honeycomb sandwich structures, respectively. In another study, the sheet-based Gyroid TPMS lattice was investigated under three-point bending test to evaluate its flexural modulus and strength. The specimens were printed using PLA polymer with various infill densities of 20%, 50% and 80%, where infill density is a process parameter which defines the density of a base material filled in a polygon. Silva et al. [139] showed that the highest flexural properties of sheet-based Gyroid TPMS lattices can be obtained when printing with a 0∘ raster angle. Results found indicate that the apparent flexural modulus and strength increase with the increase in infill density. Nevertheless, the specific relative density of the Gyroid used or the mass of the specimen were not reported. Compared to the uniaxial compression tests, there is a lack of bending tests on various polymeric metamaterials, especially the TPMS, strut-based and plate lattices, and the existing studies that perform these tests do not investigate a range of densities of these metamaterials.

### 4.3. Impact Tests

**Figure 19:**
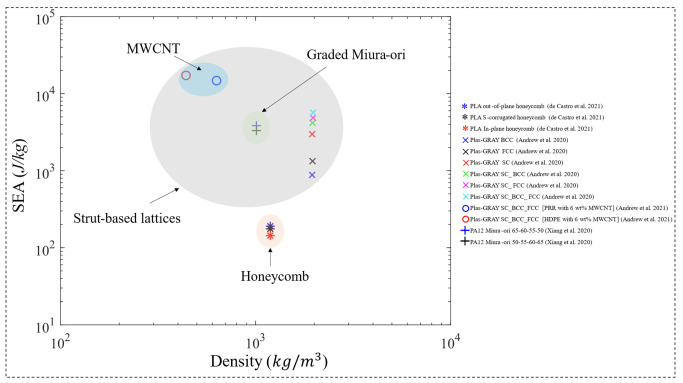
Specific energy absorption versus metamaterial density from impact physical tests on various polymeric metamaterials [40,79,137,140].

In order to quantify the amount of energy absorbed by a material during a fast collision (in a scale of milli-seconds), one of the common mechanical tests performed is the impact test. This gives a measure of a material’s toughness which is an important parameter in various applications. De Castro et al. [137] performed a Charpy impact test on similar PLA honeycomb sandwich structures that were used for three-point bending, with the three different core designs: hexagonal honeycombs oriented (i) out-of-plane and (ii) in-plane, and S-shape corrugated. With a similar mass for all three samples, the results showed that energy absorption of the out-of-plane oriented and S-shape corrugated honeycomb structures was similar and higher than the in-plane oriented honeycomb sandwich structure (Figure 19). On the other hand, Xiang et al. [140] conducted impact tests using a split Hopkinson pressure bar system on uniform and graded polymeric Miura-origami metamaterials, printed using PA12 fine grain. Miura-origami is a typical subset of open-cell rigid origami structures, where the latter is a branch of origami in which hinges replace the folding creases, and the surrounding surfaces cannot be bent or stretched during the folding process. Convex and concave creases connect the four identical parallelograms that make up a typical Miura-origami cell, where every four creases intersect at a single vertex. Different specimens made by stacking four origami sheets (6 by 9 cells in each sheet) with a 1 mm cell wall thickness were printed with different angles, referring to the angles between the two sides of a parallelogram. The range of angles considered were 50°, 55°, 60° and 65°, where uniform specimens, i.e., constant angle between all sides of the parallelogram, and graded specimens, i.e., different angles between the sides of the parallelogram, were manufactured. In terms of the uniform specimens, with an identical gas pressure used to apply the impact, results show that the peak force of the specimen with uniform 65° was greater than the specimen with uniform 55° angle. In terms of the graded specimens, it was found that the specimens with a negative gradient, i.e., the angles vary from 65° to 50° from the top sheets to the bottom, absorbed more deformation energy than the specimens with a positive gradient, i.e., the angles vary from 50° to 65° from the top sheets to the bottom. These results are included in Figure 19. In addition, the quasi-static responses of these structures were investigated using finite element simulations, with the plastic material data obtained from a tensile test of a solid specimen. Results indicate that the difference between the FEA results and experimental results during the whole deformation process was less than 10%, indicating a good agreement.

Furthermore, low-velocity impact tests have been performed on plate lattices. Andrew et al. [79] conducted impact tests on polymeric plate lattices printed with thermoplastic using SLS printing technology. Six types of core topologies were used which included SC, BCC and FCC and the three hybrid structures of SC-BCC, SC-FCC and SC-BCC-FCC (Figure 15g). All plate specimens were made of 2 × 2 × 2 number of unit cells with 16 mm unit cells. The study focused on two specimen categories which were plate-lattice specimens with constant relative density and constant plate thickness. For the former case, the relative density was set to be 35% while the thickness of the plates in different architectures varied depending upon the volume of the plates in each lattice. For the latter case, the plate thickness of all specimens was set at 1 mm while the relative density varied between the specimens. From the low velocity impact tests carried out on the different constant thickness specimens, the specimens were ranked from the highest energy absorbed to the lowest as follows SC-BCC-FCC > SC-FCC > SC-BCC > FCC > BCC > SC. From the low-velocity impact tests carried out on the different constant relative density specimens, the specimens were ranked from the highest energy absorbed to the lowest as follows SC-BCC-FCC > SC-FCC > SC-BCC > SC > FCC > BCC, as shown in Figure 19. Similarly, the same low-velocity impact tests were conducted on polypropylene random copolymer (PPR) with MWCNT plate lattices (PPR/MWCNT) and high-density polyethylene thermoplastic (HDPE) with MWCNT plate lattices (HDPE/MWCNT) [40]. The plate lattices were manufactured using the FDM printing technique with different weight percentages of MWCNTs (0, 4, 6 and 8 wt%) having an average outer diameter of 10–12 nm. The topology considered was the same as the one found to have the highest energy absorption in [75], i.e., SC-BCC-FCC hybrid topology, having a constant relative density (36%). Some of the important findings indicate that the addition of MWCNTs into the polymer matrices leads to improvement in the absorbed energy by the specimen. However, once the MWCNT weight percentage exceeded 6 wt%, the impact performance gradually reduced for both PPR/MWCNT and HDPE/MWCNT specimens, which according to the authors [40], is due to the instability of MWCNT in the polymer melt at high loadings that results in poor printing quality. At a similar MWCNT wt%, it is found that the HDPE/MWCNT specimens have a much higher energy absorption than the PPR/MWCNT specimens. However, when compared to the neat polymer lattices (0 wt% of MWCNT) the improvement in absorbed energy of HDPE/MWCNT SC-BCC-FCC plate lattices is higher than the PPR/MWCNT SC-BCC-FCC plate lattices at 2 and 4 wt% MWCNT, while it is lower than the PPR/MWCNT at 6 wt% MWCNT due to higher nucleating effect of MWCNTs in PPR matrix compared to that in HDPE matrix. The results of 6 wt% MWCNT are shown in Figure 19.

All the results of the specific energy absorption reported by the previously discussed studies are plotted in terms of the densities of the metamaterials, whenever the density is either reported or can be calculated, as presented in Figure 19. It is interesting to note that even at a lower density than others, the Plas-GRAY (Asiga company, Alexandria, Australia) SC-BB-FCC/MWCNT structures (made from PPR and HDPE) have a higher energy absorption than the other metamaterials. On the other hand, the PLA honeycomb structures show a relatively lower energy absorption than the rest. Similar to the bending tests, there is a lack of impact tests on various polymeric metamaterials, especially the TPMS and plate lattices, and the existing studies that perform these tests do not investigate a range of densities of these metamaterials, as clearly shown in Figure 19.

### 4.4. Other Tests of Polymeric Metamaterials

Looking away from the uniaxial compression, bending and impact tests, there is a scarcity of mechanical tests of polymeric metamaterials. Although there have been attempts to numerically model the mechanical behavior of TPMS structures under complex loading, such as biaxial, shear, torsion and combination of other loading types [141,142], experimental investigations would provide a clear image of the performance of these metamaterials in a variety of applications.

De Castro [137] conducted uniaxial tensile tests on the 3D sandwich structures of honeycombs (out-of-plane, S-corrugated and in-plane) and found similar results to the three-point bending tests in terms of the uniaxial modulus, where the out-of-plane honeycomb structures have the highest modulus, followed by the S-corrugated and in-plane structures. However, in terms of the ultimate strength, it is found that the S-corrugated honeycomb structures have a little higher uniaxial strength than the out-of-plane structures, followed by the in-plane structures. However, as mentioned previously, there is a clear limitation on the number of studies investigating the performance of polymeric metamaterials under various loading conditions, other than the uniaxial compression test.

## 5. Conclusions and Future Outlook 

The present review highlights the increasing interest among researchers in exploring the multi-functionality of polymeric metamaterials for diversified purposes, such as load-bearing, impact absorption, biocompatibility and heat resistance with excellent mechanical properties. A collection of recent research on the design, fabrication, testing and modeling of polymeric metamaterials was assembled in the present review. It was found that improving their fabrication procedure produces structures with maximal performance capabilities. It is anticipated that the recent advancements in designing lattice materials using machine learning tools would offer novel opportunities to enhance their manufacturing fidelity based on artificial intelligent algorithms. 

Although several lattice architectures have been proposed in the literature with the sole purpose of meeting the desired engineering function, there are still aspects related to metamaterial designs that are yet to be explored to the best of the authors’ knowledge. Often, strut-based, plate-based and comb-based lattices are derived explicitly using CAD tools which is a time-consuming process. It will be interesting to be able to construct these classes of lattice materials implicitly as the mathematically derived TPMS lattices to accelerate their designing and facilitate functional grading of their topological properties. In addition, given the major and rapid advancements in additive manufacturing, there is an increased demand for using such metamaterials in applications that do not involve uniaxial compression loading only. There is a scarcity of investigations on the mechanical properties in loading conditions such as uniaxial tension, biaxial, shear, torsion and a combination of such loading conditions, which is required to have a clear image of the performance of these polymeric metamaterials in a variety of applications. This is needed to speed up the implementation of additive manufacturing into various applications. It is important not to forget the role of machine/deep learning in filling the current gaps in the additive manufacturing process. For example, machine learning can solve the problem of assigning the best printing parameters to a printing job, which is one of the most important aspects of having a successful and functional printed component. Once enough data are generated and a proper database is set up, these printing parameters will no longer be of concern for new operators. Thus, the addition of machine learning to the additive manufacturing process can play a major role in bringing us one step closer to a world where additive manufacturing can be easily implemented in various domains with utmost accuracy.

## Figures and Tables

**Table 1:** Application of lattice materials and description of the investigated topologies and physical properties.

Study	Application	Lattice Material Topology	Base Material	Physical Property
Papetti et al. [7]	Automotive	Kelvin, cubic, octet lattices	Ceramic (Al_2_O_3_)	Mass transfer properties
Yin et al. [8]		Pyramidal structure	Fiber reinforced composites	Impact mitigation
Xiong et al. [12]	Dentistry	Porous scaffold	Ti-6Al-4V alloy	Fatigue behavior and osteointegration
Cosma et al. [16]		Body-centered cubic and circle intersections	316L stainless steel	Mechanical strength
Oladapo et al. [17]		Cubic-octahedron and Gyroid	Polyether ether ketone (PEEK) and calcium hydroxyapatite composite (cHAP)	Elastic moduli
Oladapo et al. [10]	Bone implant	Cubic-octahedron and Gyroid	PEEK-cHAP, PEEK-reduced graphene oxide	Biocompatibility
Reyes et al. [11]		Modified honeycomb	Polycaprolactone	Mechanical strength and stiffness
Li et al. [13]	Spacecraft systems	Pyramidal truss	Carbon fiber-reinforced polymer	Thermal expansion, compression and shear behavior

**Table 2:** Material fabrication details of polymeric metamaterials.

Study	AM Technique	Fabrication Challenge	Fabrication Solution
He et al. [100]	DLP	Strut deformation of polymer-derived ceramic metamaterials during the pyrolysis process	Introduction of 20 wt.% hydroxyl silicone oil prevented the deformation of struts with 0.5 mm thickness
Zhao et al. [92]	FDM	Polymeric metamaterials with high ceramic yield are so brittle to be fabricated using FDM	Improving the formability and printability of polycarbosilane using ≤5 wt.% of polypropylene
Chen et al. [111]	Micro-SLA	Cross-contamination between two different feedstocks during multi-material additive manufacturing	Integrating a self-cleansing robotic dispenser into the 3D printer for cleaning residue monomer at each layer before a new feedstock is perfused
Wang et al. [87]	FDM	Bond and joint failure between a corrugated core and face sheet panels in corrugated structures	Fabricating the structures using single-stroke integrated manufacturing for strengthening the connection between the core and the face sheet panels
		Shape retention problem during post curing procedure	Utilization of liquid deposition modeling to deposit silicon rubber between the gaps of the unit cells
Clarkson et al. [96]	DIW	Limited number of commercial printing inks with certain viscosity constrains and shear-thinning requirement	Modifying the conventional DIW procedure to expand the range of printable materials through including UV-assisted reactants in the slurry
Essmeister et al. [115]	SLA	Cracks appearing when printing millimeter scale lattice structures made of SiOC PDC	Incorporating SiC particulates within SiOC matrix to produce crack-free millimeter scale features
Verma et al. [116]	MJF	Powder entrapment zones in plate/shell-based lattice metamaterials	Introducing a honeycomb shaped structure with ventilated holes to eliminate power entrapment

## Data Availability

Not applicable.
